# Normalization of trophoblast mTOR signaling rescues impaired function in primary human trophoblast cells isolated from pregnancies complicated by fetal growth restriction

**DOI:** 10.1038/s41420-025-02801-5

**Published:** 2025-11-07

**Authors:** Ellen C. Francis, Hiroshi Shimada, Theresa L. Powell, Kristen E. Boyle, Dana Dabelea, Thomas Jansson, Fredrick J. Rosario

**Affiliations:** 1 Department of Biostatistics and Epidemiology, Rutgers School of Public Health, Piscataway, NJ USA; 2 Division of Reproductive Sciences, Department of Obstetrics and Gynecology University of Colorado, Anschutz Medical Campus, Aurora, CO USA; 3 Departments of Obstetrics & Gynecology, Sapporo Medical University, Sapporo, Japan; 4 Section of Neonatology, Department of Pediatrics, University of Colorado Anschutz Medical Campus, Aurora, CO USA; 5 Section of Nutrition, Department of Pediatrics, University of Colorado Anschutz Medical Campus, Aurora, CO USA; 6 The Lifecourse Epidemiology of Adiposity and Diabetes (LEAD) Center, Aurora, CO USA; 7 Department of Epidemiology, Colorado School of Public Health, and Department of Pediatrics, University of Colorado Anschutz Medical Campus, Aurora, CO USA

**Keywords:** Drug development, Paediatric research, Translational research

## Abstract

Fetal growth restriction (FGR) is associated with inhibition of placental mTOR signaling and amino acid transport. mTOR is a positive regulator of amino acid transport mediated by controlling the plasma membrane trafficking of SNAT2, a System A amino acid transporter isoform, and LAT1 an isoform involved in System L amino acid transport. Inhibition of mTOR complex 1 decreases SNAT2 and LAT1 plasma membrane trafficking by activating of Nedd4-2, an E3 ubiquitin ligase, and inhibition of mTOR Complex 2 decreases the protein expression of Cdc42 which limits transporter trafficking to the plasma membrane. We isolated human primary trophoblast (PHT) cells from FGR placentas and demonstrate that they maintain the in vivo FGR phenotype with increased expression of DEPTOR, an endogenous inhibitor of mTOR, reduced mTOR signaling, increased Nedd4-2 expression, lower expression of Cdc42, and decreased SNAT2 and LAT 1 protein expression in the plasma membrane, and decreased System A and L activity. We silenced *DEPTOR* in FGR PHT cells using siRNA and found normalized mTOR signaling, Nedd4-2 and Cdc42 protein expression, SNAT2 and LAT1 plasma membrane trafficking and System A and L amino acid transport activity. We also show that hypoxia induces DEPTOR upregulation in PHT cells. In the Healthy Start Study, a longitudinal pre-birth cohort, placental DEPTOR expression was correlated with lower birth weight percentile and with higher systolic and diastolic blood pressure in children at 4–6 years of age. Together, our studies provide mechanistic and translational insight into how placental DEPTOR may serve as potential mediator of fetal growth and long-term health risk. We identify a mechanistic link between increased trophoblast DEPTOR expression in FGR and decreased placental mTOR signaling and amino acid transport. Intervention strategies aimed at normalizing trophoblast mTOR signaling may be effective to improve trophoblast nutrient transport and fetal growth in FGR.

## Introduction

Fetal growth restriction (FGR) is a serious pregnancy complication affecting ~5–10% of infants worldwide [1]. FGR increases the risk for perinatal complications [1] and predisposes the individual to develop obesity, diabetes, and cardiovascular disease in childhood and adulthood [2]. However, the underlying mechanisms causing abnormal fetal growth are still not fully understood, and no specific treatment to improve fetal growth in utero is currently available. Fetal growth is significantly influenced by nutrient availability, which is associated with placental nutrient transport capacity. FGR in women is associated with reduced placental amino acid transport capacity [3, 4]. Importantly, down-regulation of key placental amino acid transport systems precedes the development of FGR in rodents [5] and non-human primates [6]. Therefore, the downregulation of placental nutrient transport may be a primary event, directly contributing to FGR rather than being in response to reduced fetal demand. Conversely, in fetal overgrowth, the activity of placental amino acid transporters reportedly increased [7, 8]. In general agreement with this concept, we recently reported that placental specific knockdown of SNAT2 amino acid transport caused FGR in mice [9] and placental-specific overexpression of LAT1 amino acid transporters resulted in fetal overgrowth [10].

Mechanistic target of rapamycin (mTOR) signaling is a highly conserved regulator of cellular metabolism, growth, and survival in response to hormones, growth factors, nutrients, energy, and stress signals [11]. mTOR forms two structurally and functionally distinct protein complexes: mTOR complex 1 (mTORC1) and mTOR complex 2 (mTORC2). mTORC1 is defined by its association with the adaptor protein Raptor (regulatory-associated protein of mTOR), while the mTORC2 complex contains Rictor (rapamycin-insensitive companion of mTOR). The role of mTOR signaling in placental function has been explored in animal models [12], ex vivo human placental tissue [8, 13], and in cultured primary human trophoblast cells [14, 15], the endocrine and transporting epithelium of the human placenta. Specifically, trophoblast mTOR is activated by insulin/IGF-1, glucose, amino acids, fatty acids, and folate and inhibited by glucocorticoids, adiponectin, hypoxia, infection, and reduced uteroplacental blood flow [16]. mTOR signaling is a positive regulator of trophoblast amino acid transport [15].

Placental mTOR signaling is inhibited in association with restricted fetal growth in both human [17, 18] and animal models of FGR [5, 19, 20]. Conversely, placental mTOR signaling is activated in association with fetal overgrowth in women [8] and animal models of fetal overgrowth [21]. Based on this body of evidence, it has been proposed that trophoblast mTOR signaling represents a critical hub in the overall homeostatic control of fetal growth, with mTOR signaling modulated according to the ability of the maternal supply line to support fetal growth [22]. However, whether restoration of trophoblast mTOR signaling can rescue impaired placental function in FGR is unknown.

The System L transporter is a sodium-independent exchanger mediating the transport of large neutral, predominantly essential amino acids, including leucine [23]. System L is a heterodimer composed of a light chain, commonly known as LAT1 (*SLC7A5*) or LAT2 (*SLC7A8*), and a heavy chain, referred to as 4F2hc/CD98 (*SLC3A2*) [24]. Sodium-dependent neutral amino acid transporter 1 (SNAT1, *SLC38A1*), SNAT2 (*SLC38A2*), and SNAT4 (*SLC38A4*) isoforms of the System A transporter are all expressed in the placenta [25] and preferentially mediate the active uptake of a range of neutral non-essential amino acids, including alanine, serine, and cysteine [26]. Studies in cultured primary human trophoblast (PHT) cells have demonstrated that mTOR signaling is a positive regulator of trophoblast amino acid (System A and L), folate transport, and mitochondrial respiration [15, 27, 28]. We are utilizing cultured PHT cells from FGR placentas to demonstrate that the reduced mTOR, lower SNAT2 and LAT1 transporter abundance in MVM and reduced system A and L amino acid transporter activity that was previously found in ex vivo placental tissues [17, 18] is retained in cultured PHT cells isolated from FGR pregnancy. By using these phenotypically consistent cultured primary human trophoblast cells, we can investigate the role of DEP domain-containing mTOR interacting protein (DEPTOR) in the downregulation of mTORC1 and mTORC2 signaling in FGR placentas.

DEPTOR is an endogenous inhibitor of mTORC1 and mTORC2 signaling [29] and plays an essential role in various cellular processes, including cell growth, apoptosis, autophagy, and adipogenesis [30]. Recently, it was shown that the reduction of DEPTOR expression promotes muscle hypertrophy and increased myoblast size [31]. We recently demonstrated that activation of mTOR by silencing of DEPTOR in primary human trophoblast cells isolated from healthy term placentas resulted in a marked increase in System A and L amino acid transport activity [27, 28]. Therefore, it is possible that DEPTOR silencing represents an approach to improve placental function, including amino acid transport in FGR. FGR is frequently associated with placental hypoxia [32], which contributes to oxidative stress [33], impaired nutrient transport [34], and disrupted placental signaling pathways [35], including mTOR. DEPTOR is a negative regulator of mTOR signaling; however, its upstream regulation in the placenta remains poorly understood. Furthermore, to expand the clinical relevance of our studies, we examined placental DEPTOR expression in a longitudinal pre-birth cohort [36, 37] and assessed its association with birth weight percentile and blood pressure outcomes in childhood.

In the current study, we hypothesized that restoration of mTOR signaling by DEPTOR silencing in primary trophoblast cells isolated from placentas of FGR pregnancies improves trophoblast amino acid uptake, that low oxygen tension induces DEPTOR expression in trophoblasts, and that placental DEPTOR expression is associated with long-term outcomes in children.

## Results

### FGR PHT cells retain the phenotype described in FGR placental tissue

**Fig. 1:**
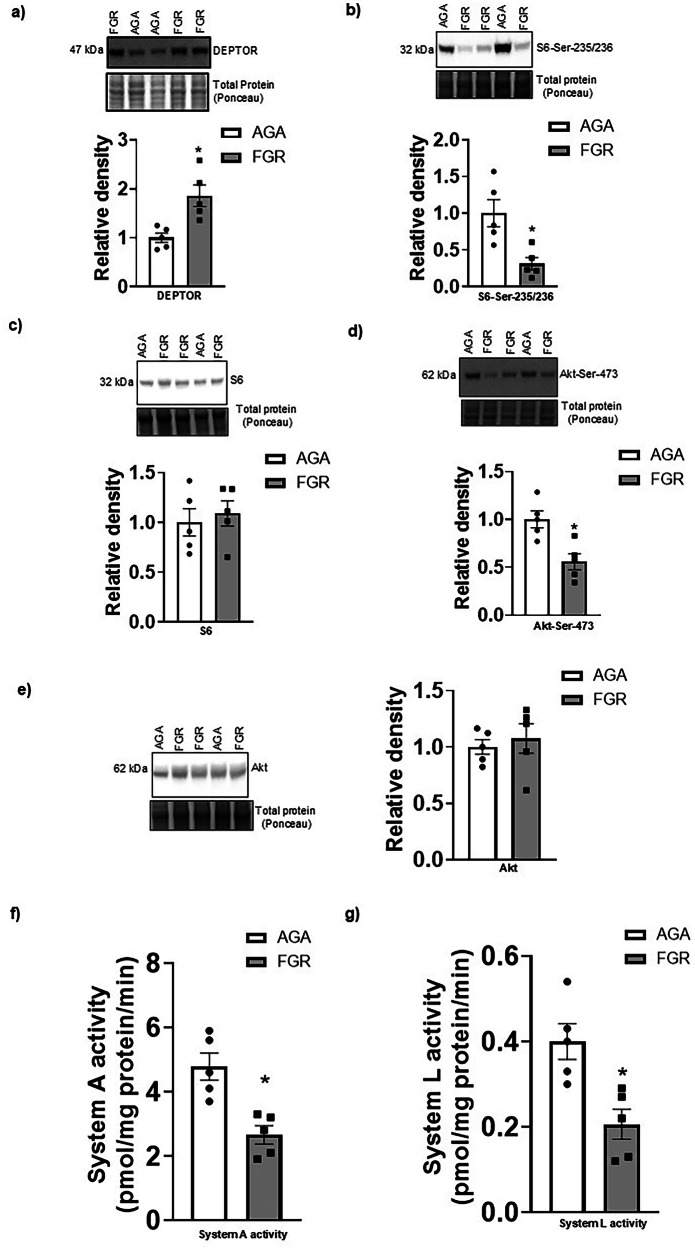
Elevated DEPTOR protein expression associated with decreased mTORC1, mTORC2 signaling and System A and System L amino acid transport activity in FGR PHT cells. Representative western blots for **a** DEPTOR, **b** S6 (Ser-235/236), **c** S6, **d** Akt (Ser-473) and **e** Akt in cell lysates of AGA and FGR PHT cells. Histogram summarizes the Western blotting data. Equal loading was performed. After normalization to total protein, the mean density of AGA samples was assigned an arbitrary value of 1. **f**, **g** System A activity was measured as the Na^+^-dependent uptake of [^14^C] MeAIB and System L activity was determined as BCH-inhibitable uptake of [^3^H] leucine. System A (**f**) and System L (**g**) transporters activity were lowered in FGR primary human trophoblast cells. Values are given as means  ± sem. *P < 0.05 vs. AGA, *n* = 5 each group, Student’s *t* test.

We determined the DEPTOR protein level in AGA and FGR PHT cells. As shown in Fig. 1a, DEPTOR protein expression was significantly higher in FGR PHT cells (+95%, *n* = 5/group, *p* = 0.009) as compared to AGA PHT cells. In addition, we also measured the phosphorylation state of the mTORC1 and mTORC2 substrates, S6 and Akt, respectively in PHT cells. Phosphorylation of S6 at Serine 235/236 was significantly decreased (−69%, *n* = 5/group, *p* = 0.009) in PHT cells isolated from FGR placentas (Fig. 1b). However, the total S6 expression (*n* = 5/group, *p* = 0.645) was comparable between AGA and FGR groups (Fig. 1c). Moreover, Akt phosphorylation at Ser-473 was lower in FGR PHT (−44%, *n* = 5/group, *p* = 0.007) cells as compared to AGA (Fig. 1d). In contrast, total Akt expression was not different between AGA and FGR PHT cells (*n* = 5/group, *p* = 0.62, Fig. 1e). Taken together, this data indicates that elevated DEPTOR expression in FGR PHT cells is associated with decreased functional downstream targets of mTORC1 and mTORC2 signaling.

### Decreased System A and System L transport activity in FGR PHT cells

We utilized radiolabeled ^14^C methyl-aminoisobutyric acid (MeAIB) to measure System A amino acid transport activity. As shown in Fig. 1f, System A activity was significantly lower in FGR PHT cells (−44%, *n* = 5/group, *p* = 0.003) as compared to AGA PHT cells. In addition, System L amino acid transport activity was also significantly reduced (−49%, *n* = 5/group, *p* = 0.008) in the FGR PHT cells (Fig. 1g).

### Normalizing DEPTOR protein levels in FGR PHT cells restores mTORC1 and mTORC2 signaling

**Fig. 2:**
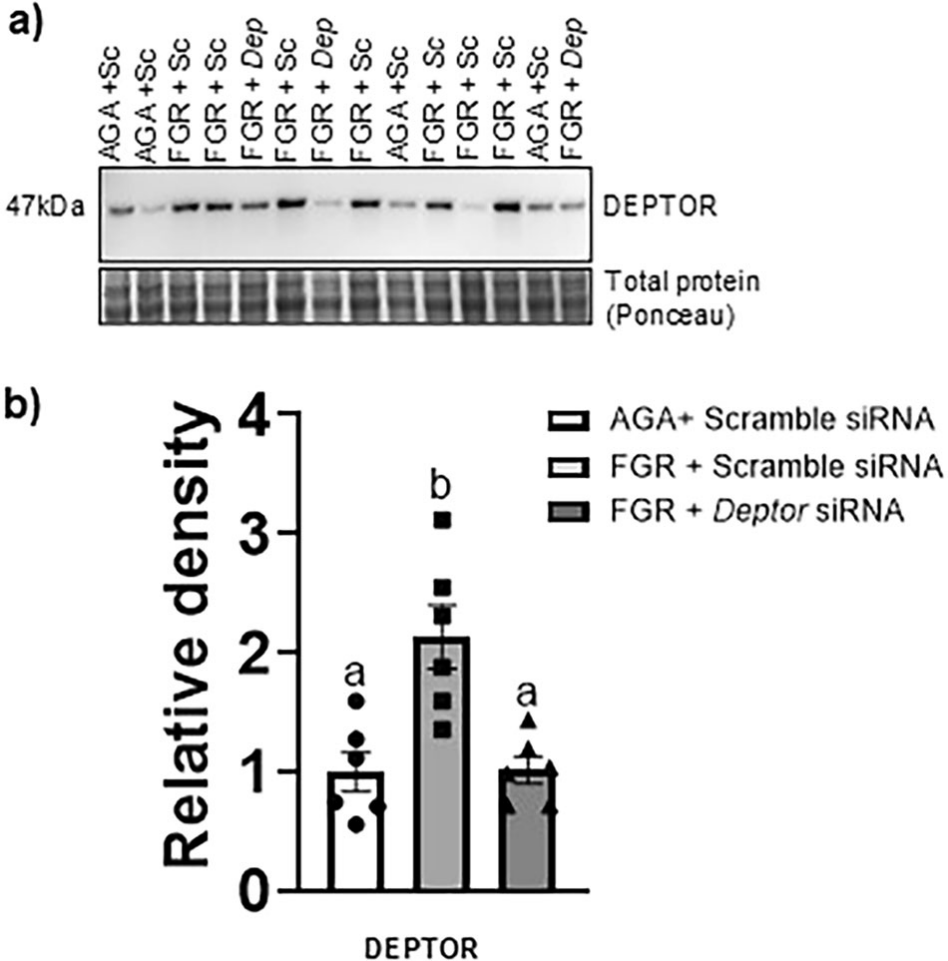
Effect of *DEPTOR* siRNA silencing on DEPTOR protein expression in FGR PHT cells. **a** Representative Western blot for DEPTOR in cell lysates of AGA and FGR PHT cells transfected with either scramble or *DEPTOR siRNA*. **b** Histogram summarizes the Western blotting data. Equal loading was performed. After normalization to total protein, the mean density of AGA samples was assigned an arbitrary value of 1. Values are given as means  sem. *P < 0.05 vs. AGA PHT cells transfected with scramble siRNA; Means without a common letter differ significantly (*P*  < 0.05) by One-way ANOVA with Tukey–Kramer multiple comparisons post hoc test.

**Fig. 3:**
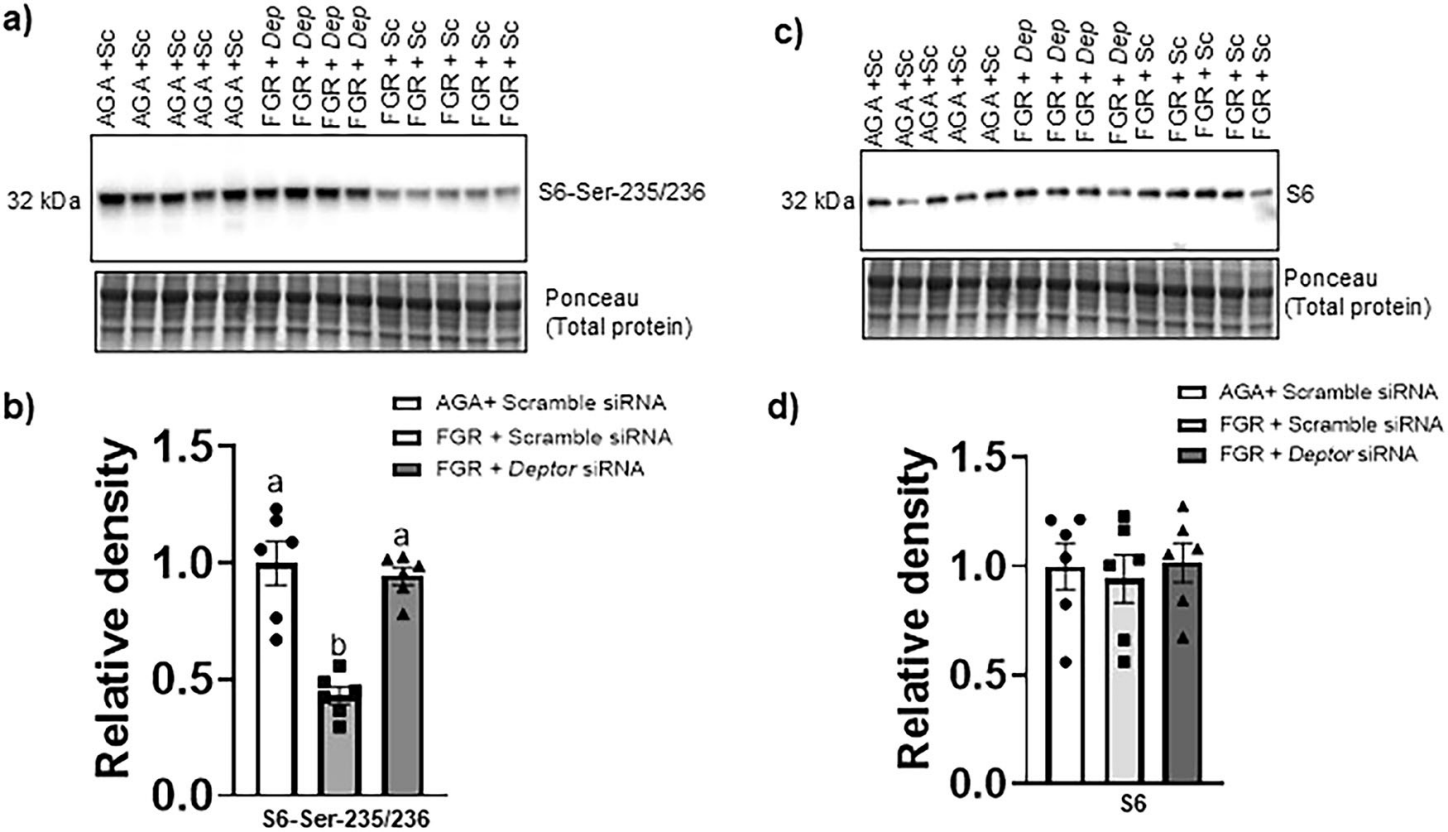
Normalizing DEPTOR protein levels in FGR PHT cells restores the mTORC1 signaling. **a**, **c** Representative Western blot for S6-Ser 235/236 and total S6 in cell lysates of AGA and FGR PHT cells transfected with either scramble or *DEPTOR siRNA*. **b**, **d** Histogram summarizes the Western blotting data. Equal loading was performed. After normalization to total protein, the mean density of AGA samples was assigned an arbitrary value of 1. Values are given as means ±  sem. *P < 0.05 vs. AGA PHT cells transfected with scramble siRNA; Means without a common letter differ significantly (*P*  < 0.05) by One-way ANOVA with Tukey–Kramer multiple comparisons post hoc test.

**Fig. 4:**
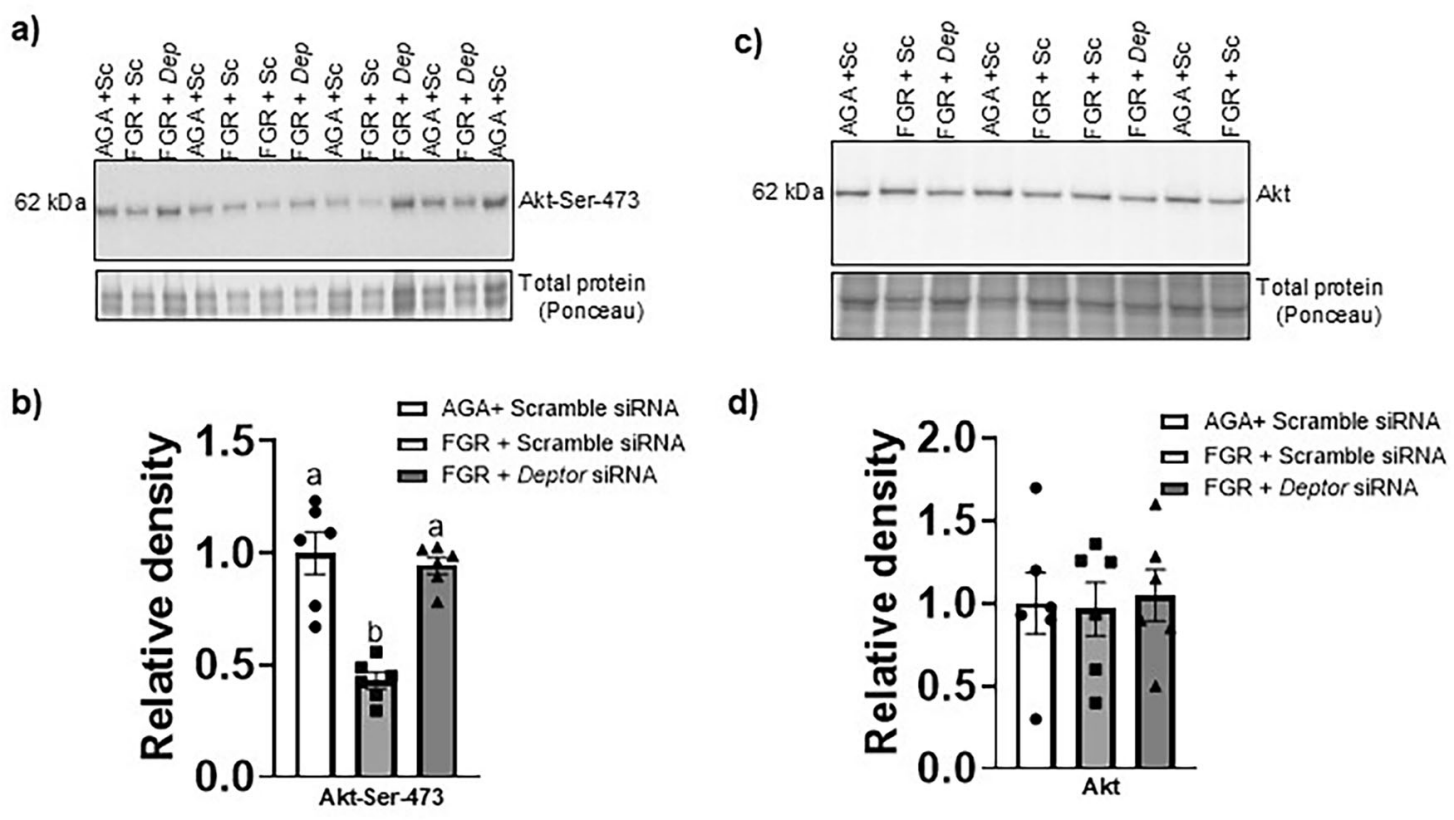
Normalizing DEPTOR protein levels in FGR PHT cells restores the mTORC2 signaling. **a**, **c** Representative Western blot for Akt-Ser 473 and total Akt in cell lysates of AGA and FGR PHT cells transfected with either scramble or *DEPTOR siRNA*. **b**, **d** Histogram summarizes the Western blotting data. Equal loading was performed. After normalization to total protein, the mean density of AGA samples was assigned an arbitrary value of 1. Values are given as means ±  sem. *P < 0.05 vs. AGA PHT cells transfected with scramble siRNA; Means without a common letter differ significantly (*P*  < 0.05) by One-way ANOVA with Tukey–Kramer multiple comparisons post hoc test.

To determine if the elevated expression of DEPTOR in FGR PHT cells is a potential regulator of mTOR downstream targets, we normalized the DEPTOR protein levels in FGR PHT cells using *DEPTOR* siRNA. Silencing of *DEPTOR* in FGR PHT cells (Fig. 2a, b) lowered the DEPTOR protein level in FGR PHT cells to the level of AGA PHT cells (*n* = 6/group, *p* = 0.0009). Normalization of *DEPTOR* levels in FGR PHT cells rescued the decreased phosphorylation of both S6 ribosomal protein (*n* = 6/group, *p* = 0.0001) at Serine 235/236 (mTORC1, Fig. 3a, b) and Akt at Serine 473 (mTORC2, *n* = 6/group, *p* = 0.002) in FGR PHT cells (Fig. 4a, b). Total S6 (*p* = 0.868, *n* = 6/group, Fig. 3c, d) and Akt (*p* = 0.944, *n* = 6/group, Fig. 4c, d) protein expression levels were comparable between groups.

### Normalizing DEPTOR protein level in FGR PHT cells restores normal System A and System L amino acid transport activity

**Fig. 5:**
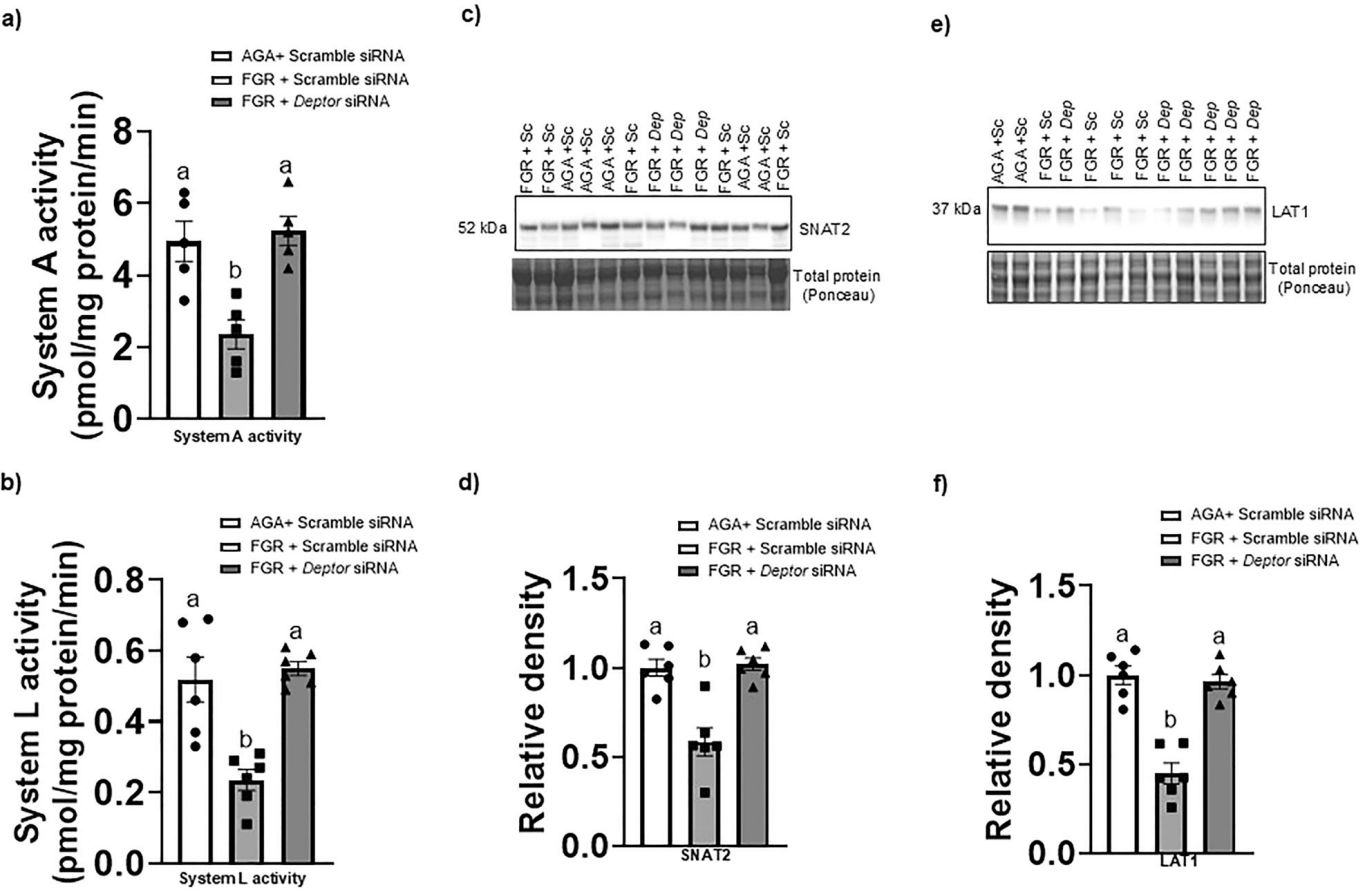
Normalizing DEPTOR protein level in FGR PHT cells restores System A and System L amino acid transport activity and microvillus plasma membrane SNAT2 and LAT1 expression. **a**, **b** System A activity was measured as the Na^+^-dependent uptake of [^14^C] MeAIB and System L activity was determined as BCH-inhibitable uptake of [^3^H] leucine. System A and System L transporter activity were measured in AGA and FGR PHT cells transfected with either scramble or *DEPTOR siRNA*. **c**, **e** Representative Western blots for MVM SNAT2 and LAT1 of AGA and FGR PHT cells transfected with either scramble or *DEPTOR siRNA*. **d, f** Histogram summarizes the Western blotting data. Equal loading was performed. After normalization to total protein, the mean density of AGA samples was assigned an arbitrary value of 1. Values are given as means ±  sem. *P < 0.05 vs. AGA PHT cells transfected with scramble siRNA; Means without a common letter differ significantly (*P*  < 0.05) by One-way ANOVA with Tukey–Kramer multiple comparisons post hoc test.

To determine whether the effects of *DEPTOR* normalization in FGR PHT cells on mTORC1/2 signaling were physiologically significant, we measured System A and System L amino acid transport activity in AGA and FGR PHT cells transfected with scramble and/or *DEPTOR* siRNA. Normalization of DEPTOR levels in FGR PHT cells restored System A (*n* = 6/group, *p* = 0.001, Fig. 5a) and System L (*n* = 6/group, *p* = 0.0001, Fig. 5b) mediated amino acid transport activity to the levels that are comparable to AGA PHT cells.

### Normalizing DEPTOR protein level in FGR PHT cells restores the microvillus plasma membrane SNAT2 and LAT1 expression

We have previously demonstrated that mTORC1/C2 signaling regulates System A and System L transporter activity mediated by posttranslational mechanisms, involving the control of the plasma membrane trafficking of System A transporter isoform SNAT2 and System L amino acid transporter isoform LAT1 [12, 14, 15]. To study changes in transporter trafficking of SNAT2 and LAT1 in FGR and AGA PHT cells, we isolated the microvillous plasma membrane (MVM) fraction from cultured PHT cells and measured the MVM protein expression of SNAT2 and LAT1. Importantly, MVM SNAT2 and LAT1 expressions were significantly lower in the FGR PHT cells as compared to the AGA PHT cells (Fig. 5c–f). However, normalization of DEPTOR protein levels in FGR PHT cells restored the MVM SNAT2 (*n* = 6/group, *p* = 0.0002) and LAT1 (*n* = 6/group, *p* = 0.0001) expression to the level comparable of AGA PHT cells (Fig. 5c–f). These findings are consistent with the demonstration that trophoblast mTORC1 and mTORC2 regulates System A and System L in cultured primary human trophoblast cells by modulating the plasma membrane trafficking of SNAT2 and LAT1, respectively [15].

### Normalizing DEPTOR protein levels in FGR PHT cells restores Nedd4-2 expression

**Fig. 6:**
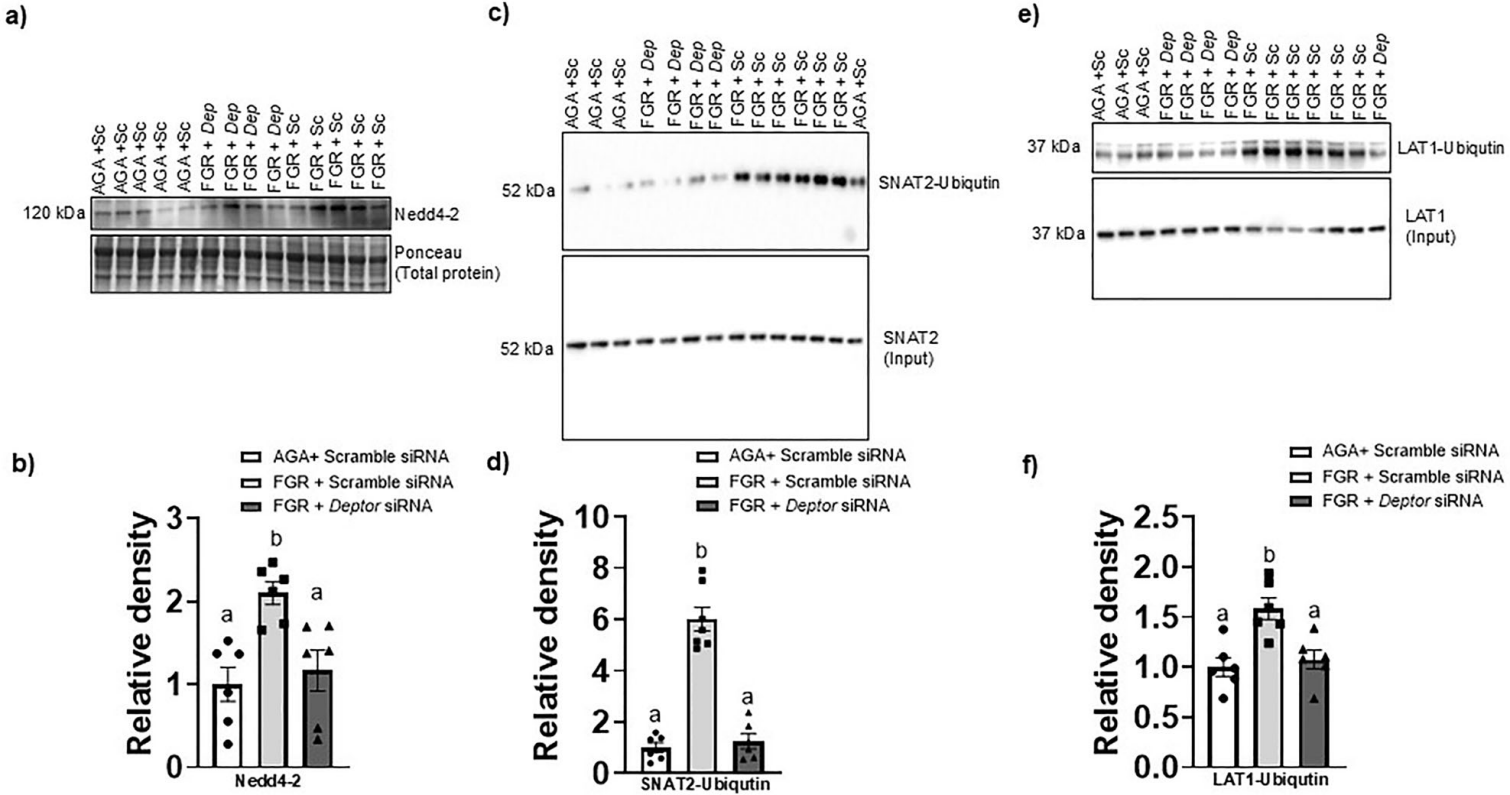
Normalizing DEPTOR protein level in FGR PHT cells restores Nedd 4-2 expression and prevents Nedd4-2 mediated ubiquitination of the SNAT2 and LAT1. **a**, **b** Representative Western blot for Nedd4-2 in cell lysates of AGA and FGR PHT cells transfected with either scramble or *DEPTOR siRNA*. **c**–**f** Decreased ubiquitination of SNAT2 and LAT1 in response to normalizing DEPTOR protein level in FGR PHT cells. Cell lysates from AGA and FGR PHT cells transfected with either scramble or *DEPTOR siRNA* were used for immunoprecipitation (IP) with an anti-SNAT2 (**c**, **d**) or LAT1 antibody (**e**, **f**). Immunoprecipitated proteins were separated by SDS-PAGE and blotted with an anti-ubiquitin (Ub) antibody. **b, d, f** Histogram summarizes the Western blotting data. Equal loading was performed. After normalization to total protein, the mean density of AGA samples was assigned an arbitrary value of 1. Values are given as means ±  sem. *P < 0.05 vs. AGA PHT cells transfected with scramble siRNA; Means without a common letter differ significantly (*P*  < 0.05) by One-way ANOVA with Tukey–Kramer multiple comparisons post hoc test.

We have shown that mTORC1 inhibition increases the protein expression of Nedd4-2, which is one of the E3 ubiquitin ligases that catalyzes ubiquitination of plasma membrane proteins and controls cell surface expression of SNAT2 and LAT1 transporters in PHT cells [28]. We found that Nedd4-2 expression (+110%, *n* = 6/group, *p* = 0.004, Fig. 6a, b) was higher in FGR PHT cells transfected with scramble siRNA as compared to PHT cells transfected with scramble siRNA. However, normalization of DEPTOR protein levels in FGR PHT cells restored Nedd4-2 protein expression (*n* = 6/group, *p* = 0.003) to the level comparable of AGA PHT cells (Fig. 6a, b), suggesting that DEPTOR-mTOR signaling regulates the membrane trafficking of the SNAT 2 and LAT1 in FGR PHT cell mediated by Nedd4-2 dependent ubiquitination.

### Normalizing DEPTOR protein level in FGR PHT cells prevents increased ubiquitination of SNAT2 and LAT1

Ubiquitination is a post-translational modification that has been shown to be associated with the regulation of the plasma membrane expression of SNAT2 and LAT1 by mTORC1 in PHT cells [14]. To study the molecular mechanisms linking elevated DEPTOR signaling to inhibition of System A and L amino acid transporter isoform intracellular trafficking in FGR PHT cells, we measured the System A (SNAT2) and System L (LAT1) transporter isoform ubiquitination in FGR PHT cell lysates following normalization of DEPTOR protein expression. To this effect, we immunoprecipitated proteins in the cell lysates of AGA/FGR PHT cells using anti-SNAT2 or LAT1 antibodies. The proteins in the immunoprecipitates were then separated by SDS-PAGE and subsequently immunoblotted with an anti-ubiquitin antibody. As shown in Fig. 6c–f, normalization of DEPTOR protein levels in FGR PHT cells decreased ubiquitination of SNAT2 (*p* = 0.0001, *n* = 6-7/group, Fig. 6c, d) and LAT1 (*p* = 0.0002, *n* = 6/group, Fig. 6e, f) as compared FGR PHT cells.

### Normalizing DEPTOR protein level in FGR PHT cells restores Cdc42 expression

**Fig. 7:**
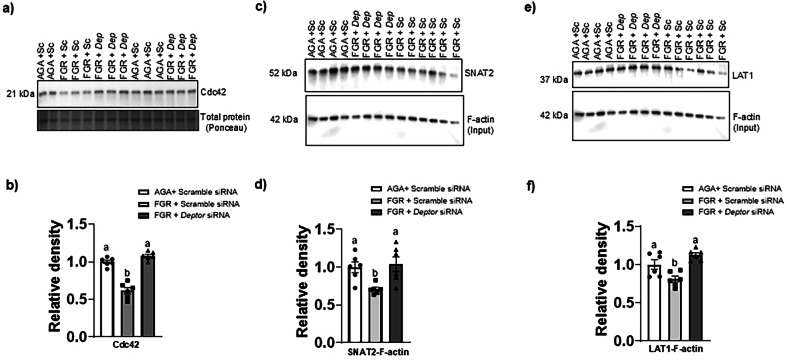
Normalizing DEPTOR protein level in FGR PHT cells restores Cdc42 expression and restores the association of F-actin with SNAT2 and LAT1. **a**, **b** Representative Western blot for Cdc42 in cell lysates of AGA and FGR PHT cells transfected with either scramble or *DEPTOR siRNA*. **c**–**f** Normalizing DEPTOR protein level in FGR PHT cells restores the association of F-actin with SNAT2 and LAT1. Cell lysates from AGA and FGR PHT cells transfected with either scramble or *DEPTOR siRNA* were used for immunoprecipitation (IP) with an anti-F actin antibody. Immunoprecipitated proteins were separated by SDS-PAGE and blotted with an anti-SNAT2 (**c**, **d**) or LAT1 antibody (**e**, **f**). Histogram summarizes the Western blotting data. Equal loading was performed. After normalization to total protein, the mean density of AGA samples was assigned an arbitrary value of 1. Values are given as means ±  sem. *P < 0.05 vs. AGA PHT cells transfected with scramble siRNA; Means without a common letter differ significantly (*P*  < 0.05) by One-way ANOVA with Tukey–Kramer multiple comparisons post hoc test.

We recently demonstrated that inhibition of mTORC2 signaling decreases the protein levels of Cdc42 and Rac1, which are among the most well characterized members of the Rho family of GTPases that regulate F-actin assembly and disassembly and selectively suppresses LAT1/SNAT2 transporter trafficking to the plasma membrane in PHT cells [27]. We measured Cdc42 protein expression in cell lysates of AGA and FGR PHT cells and found that Cdc42 (-39%, *n* = 6/group, *p* = 0.0001, Fig. 7a, b) expression was lowered in FGR PHT cells as compared to AGA PHT cells. Importantly, normalization of DEPTOR protein levels in FGR PHT cells restored Cdc42 expression (*n* = 6/group, *p* = 0.0001) to the level comparable of AGA PHT cells (Fig. 7a, b), suggesting that DEPTOR-mTOR signaling regulates the SNAT2 and LAT1 transporter trafficking in FGR PHT cell mediated by Cdc42 dependent F-actin membrane trafficking.

### Normalizing DEPTOR protein level in FGR PHT cells restores the association of F-actin with SNAT2 and LAT1

F-actin directly binds to the SNAT2 and LAT1 and translocate the transporter protein from cytosolic compartments to the plasma membrane in PHT cells [27]. To study the molecular mechanisms linking elevated DEPTOR signaling to decreased system A and L amino acid transporter isoform trafficking, we measured system A (SNAT2) and system L (LAT1) transporter isoform association with F-actin in FGR PHT cell lysates following normalization of DEPTOR protein expression. To this effect, we immunoprecipitated proteins in the cell lysate using an anti-F-actin antibody. Proteins in the immunoprecipitate were then separated by SDS-PAGE and subsequently immunoblotted with anti- SNAT2 or LAT1 antibody. As shown in Fig. 7c–f, normalization of DEPTOR protein levels in FGR PHT cells restored F-actin association with SNAT2 (*p* = 0.0001, *n* = 6/group, Fig. 7c, d) and LAT1 (*p* = 0.0002, *n* = 6/group, Fig. 7e, f) as compared to FGR PHT cells.

### Normalizing DEPTOR protein levels in FGR PHT cells restores the basal plasma membrane (BM) LAT1 expression

**Fig. 8:**
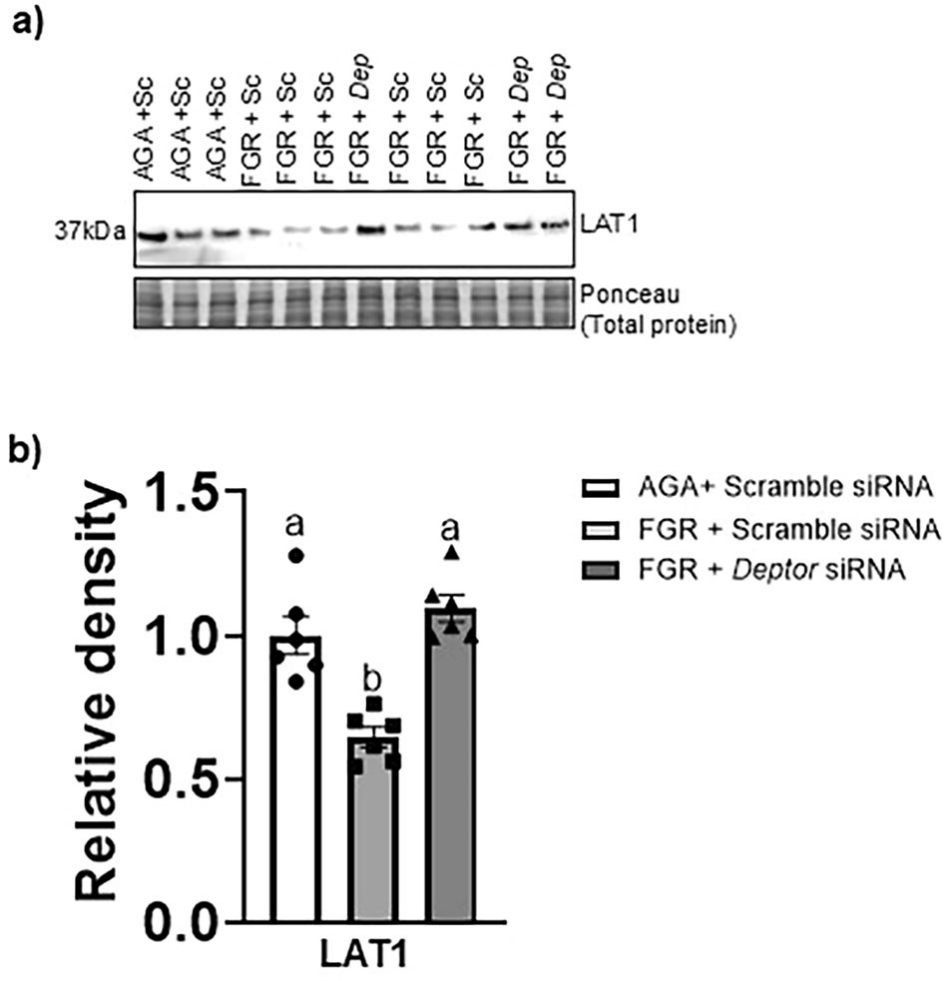
Normalizing DEPTOR protein level in FGR PHT cells restores the basal plasma membrane (BM) LAT1 expression. **a** Representative Western blots for BM LAT1 of AGA and FGR PHT cells transfected with either scramble or *DEPTOR siRNA*. **b** Histogram summarizes the Western blotting data. Equal loading was performed. After normalization to total protein, the mean density of AGA samples was assigned an arbitrary value of 1. Values are given as means ±  sem. *P < 0.05 vs. AGA PHT cells transfected with scramble siRNA; Means without a common letter differ significantly (*P*  < 0.05) by One-way ANOVA with Tukey–Kramer multiple comparisons post hoc test.

The basal System L amino acid transport activity in the placenta is significantly reduced in FGR [38]. We previously showed that Cdc42 silencing in AGA PHT cells caused a significant decrease in the expression of the System L transporter isoform LAT1 in the BM fraction [27]. In the current study, we observed decreased expression of Cdc42 in the FGR PHT cells. We investigated whether this reduced expression of Cdc42 was associated with lower levels of BM LAT1 in FGR PHT cells. To this effect, we isolated the BM from the AGA and FGR PHT cell lysates and measured LAT1 expression in the BM fraction. As shown in Fig. 8a, b, BM LAT1 expression was significantly lower in the FGR PHT cells as compared to the AGA PHT cells (−36%, *n* = 6/group, *p* = 0.0004). However, normalization of DEPTOR protein levels in FGR PHT cells restored the BM LAT1 (*n* = 6/group, *p* = 0.0001) expression to the level comparable of AGA PHT cells (Fig. 8a, b).

### Hypoxia induces DEPTOR protein expression in PHT cells

As shown in Supplemental Fig. 6, hypoxia significantly increased DEPTOR protein expression (+138%, *n* = 6/group, *p* = 0.02) in PHT cells compared to cells maintained under normoxic conditions, suggesting that low oxygen tension is a regulatory stimulus for DEPTOR upregulation in human trophoblasts. This finding is consistent with the well-documented activation of stress-responsive signaling pathways under hypoxia and aligns with the pathophysiological features of the FGR placenta, where chronic hypoxia is a hallmark. These results support the hypothesis that DEPTOR expression is responsive to environmental cues such as oxygen availability and may act as a molecular mediator linking placental hypoxia to suppression of mTOR signaling and impaired nutrient transport.

### Placental DEPTOR expression is elevated in FGR/SGA and correlates with birth weight and childhood blood pressure

**Fig. 9:**
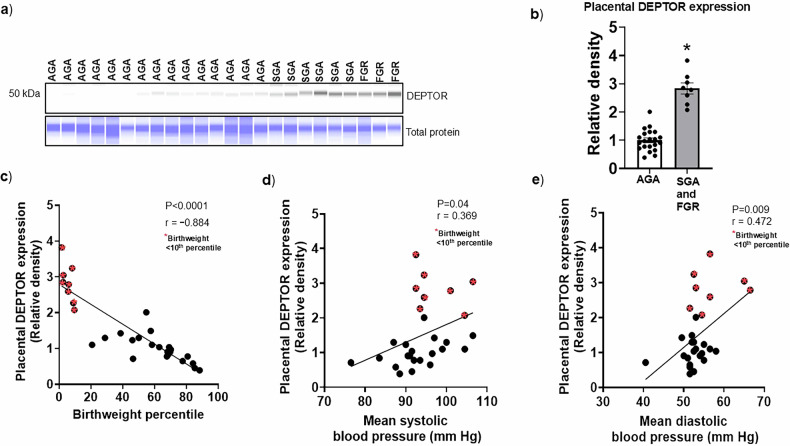
Elevated placental DEPTOR protein expression in FGR/SGA is associated with lower birth weight and higher childhood blood pressure at 4–6 years of age. **a** Representative Western Blot of DEPTOR expression in placental homogenates from Appropriate for Gestational Age (AGA), small-for-gestational-age (SGA), and fetal growth restriction (FGR) pregnancies, measured using capillary immunoblotting (Jess™ Simple Western). Total protein staining was used for normalization. **b** Placental DEPTOR protein expression is elevated in FGR and SGA pregnancies (Birth weight percentile range, 1.5–9.5). Data summarizes the Western blotting data. Quantification of DEPTOR expression in AGA (*n* = 21) and combining SGA and FGR (*n* = 8) placentas. Due to limited sample size, FGR (*n* = 3) and small-for-gestational-age (SGA, *n* = 5) cases were combined into a single group (FGR/SGA, *n* = 8), defined by a birth weight percentile range from 1.5 to 9.5 (*n* = 8). The control group (Appropriate for Gestational Age, AGA) consisted of placentas from pregnancies with birth weight percentiles ranging from 28.3 to 88.3 (*n* = 21). This stratification allowed us to assess the impact of impaired fetal growth on placental signaling and postnatal child outcomes. Equal loading was performed. After normalization to total protein, the mean density of AGA samples was assigned an arbitrary value of 1. *p < 0.05 vs. AGA, Student’s *t* test. **c** Placental DEPTOR protein expression is inversely correlated with birth weight percentile (r = −0.884, *p* < 0.0001, *n* = AGA (*n* = 21) and combining SGA and FGR (*n* = 8) placentas.). r = Pearson correlation coefficient. **d, e** Positive correlation between placental DEPTOR protein expression and systolic/diastolic blood pressure at 4–6 years of age (Systolic BP, r = 0.369, p = 0.04; Diastolic BP, r = 0.472, p = 0.009, *n* = AGA (*n* = 21) and combining SGA and FGR (*n* = 8) placentas). r = Pearson correlation coefficient.

To investigate the relationship between placental DEPTOR expression, fetal growth, and offspring blood pressure levels, we analyzed placental samples from pregnancies stratified by birth weight percentile. Due to limited sample size, FGR (*n* = 3) and small-for-gestational-age (SGA, *n* = 5) cases were combined into a single group (FGR/SGA, *n* = 8), defined by a birth weight percentile range from 1.5 to 9.5 (*n* = 8). The control group (Appropriate for Gestational Age, AGA) consisted of placentas from pregnancies with birth weight percentiles ranging from 28.3 to 88.3 (*n* = 21). This stratification allowed us to assess the impact of impaired fetal growth on placental signaling and postnatal child outcomes. Clinical characteristics of both groups are summarized in Supplementary Table 2. Associations between birth weight percentile and childhood blood pressure at 4–6 years of age are presented in Supplemental Fig. 7. As shown in Fig. 9a, b, DEPTOR expression was significantly increased in placentas from the SGA and FGR group compared to AGA controls (*p* < 0.0001; Fig. 9a, b). A strong inverse correlation was observed between placental DEPTOR expression and birth weight percentile (r = −0.884, *p* < 0.0001; Fig. 9c), indicating that elevated DEPTOR is associated with reduced fetal growth. Importantly, placental DEPTOR expression also correlated with higher systolic (r = 0.369, *p* = 0.04; Fig. 9d) and diastolic (r = 0.472, *p* = 0.009; Fig. 9e) blood pressure in children at 4–6 years of age. These findings suggest that increased placental DEPTOR may contribute not only to impaired fetal growth but also to long-term cardiovascular risk in offspring.

## Discussion

**Fig. 10:**
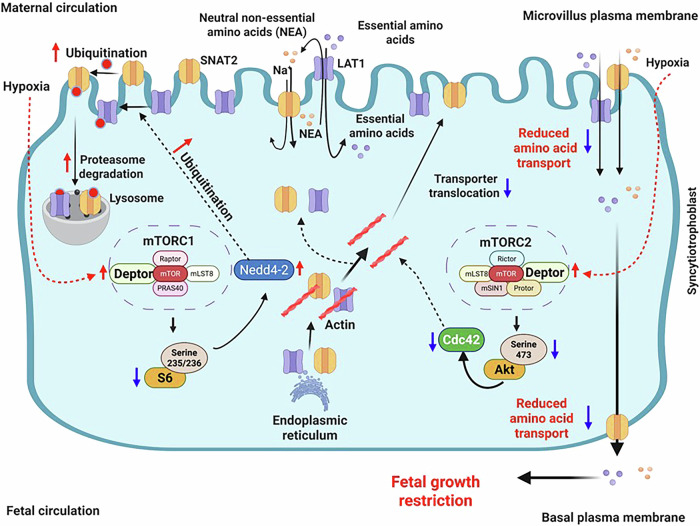
Proposed model linking elevated DEPTOR expression to impaired trophoblast mTOR signaling and reduced amino acid transport in human fetal growth restriction. The findings of the present study support a model in which trophoblast DEPTOR expression is upregulated in human FGR, leading to inhibition of both mTORC1 and mTORC2 signaling pathways. Inhibition of mTORC1 increases the ubiquitination of specific System A (SNAT2) and L (LAT1) amino acid transporter isoforms via activation of Nedd4-2, resulting in decreased transporter abundance at the syncytiotrophoblast plasma membrane and reduced placental System A and L amino acid transport activity. Simultaneously, the inhibition of mTORC2 decreases Cdc42 expression, disrupting actin cytoskeletal dynamics required for amino acid transporter trafficking, further limiting fetal amino acid availability. Hypoxia, a hallmark of FGR, contributes to elevated DEPTOR expression in trophoblast cells, linking environmental stress to mTOR suppression. These molecular events converge to impair amino acid uptake from maternal circulation and restrict nutrient delivery to the fetus, contributing to reduced fetal growth. Red arrows indicate upregulation; blue arrows indicate downregulation; red dashed lines represent hypoxia-induced effects; red text highlights key outcomes in FGR trophoblast cells.

We have successfully established that PHT cells isolated from FGR pregnancies maintain the distinct phenotype previously described in placental tissues [17, 18]. This includes down regulation mTORC1 and mTORC2 signaling activity in FGR PHT cells, which was associated with a marked down-regulation MVM SNAT 2 and LAT 1 amino acid transporter protein expression in the plasma membrane. In addition, System A and L amino acid transport activity was inhibited in PHT cells isolated from FGR pregnancies, consistent with previous reports of decreased activity of these two key amino acid transporters in syncytiotrophoblast microvillous plasma membranes isolated from human FGR placentas [3, 39]. We demonstrate for the first time that DEPTOR protein expression is higher in FGR than in AGA PHT cells. Importantly, *DEPTOR* silencing in FGR PHT cells restored mTORC1 and mTORC2 signaling activity to the levels observed in AGA PHT cells, suggesting that *DEPTOR* knockdown constitutes an effective approach to restoring mTOR signaling in the FGR placenta. Furthermore, restoration of mTORC1 and mTORC2 signaling following *DEPTOR* silencing normalized the System A and L amino acid transport activity in cultured PHT cells. SNAT2 and LAT1 isoform protein expression in MVM were also found to be similar to levels found in AGA PHT cells with *DEPTOR* knockdown in cultured FGR cells. These findings are consistent with the model that DEPTOR regulates mTORC1 and mTORC2 signaling and System A and System L amino acid transport activity in cultured primary human trophoblast cells by modulating the plasma membrane trafficking of SNAT2 and LAT1, respectively [15] (Fig. 10). Furthermore, our data reveals that hypoxia significantly induces DEPTOR protein expression in primary human trophoblast (PHT) cells. In a longitudinal pre-birth cohort study, we demonstrate that placental DEPTOR expression is significantly elevated in pregnancies complicated by fetal growth restriction (below the 10th percentile) outcomes compared to appropriate-for-gestational-age (AGA) controls. Moreover, higher DEPTOR levels were inversely correlated with birth weight percentile and positively associated with systolic and diastolic blood pressure at 4–6 years of age, suggesting a role in developmental programming of cardiovascular risk. Intervention strategies aiming at restoring placental mTOR signaling may be effective to improve placental nutrient transport and fetal growth in pregnancies at risk of FGR.

In this report, we demonstrate for the first time that mTORC1 and mTORC2 signaling is reduced in isolated trophoblast cells from human FGR pregnancy, recapitulating the findings in *ex vivo* human FGR placentas [13, 18, 40–42] and various animal models of growth restriction [5, 16, 19, 20, 43–47]. Inhibition of placental mTORC1 and mTORC2 signaling has been reported to occur before fetal growth restriction in rats fed a low-protein diet [5] and in baboons subjected to moderate calorie restriction in pregnancy [6], suggesting that changes in placental mTOR signaling are a cause rather than a secondary consequence of abnormal fetal growth. We recently demonstrated that trophoblast-specific mTOR knockdown in pregnant mice using piggyBac transposon-enhanced pronuclear injection resulted in a fetal growth restriction [12, 48] confirming a cause-and-effect relationship between placental mTOR signaling and fetal growth. Importantly, in the current study we demonstrate that normalizing the protein expression of DEPTOR in FGR PHT cells restored mTORC1 and mTORC2 signaling, consistent with the possibility that modulating DEPTOR expression would be an effective approach to restoring mTORC1 and mTORC2 signaling in the FGR placenta.

We have previously demonstrated that mTOR signaling regulates trophoblast plasma membrane SNAT2 and LAT1 expression through post translational mechanisms [14, 15, 27]. Additionally, mTORC1 and mTORC2 control SNAT2 and LAT1 membrane trafficking by distinct cellular mechanisms [21, 24]. Specifically, mTORC1 inhibition increased the expression Nedd4-2, a E3 ubiquitin ligase, which promoted SNAT2 and LAT1 ubiquitination and subsequent removal from the plasma membrane [14]. Moreover, Nedd4-2 silencing increased the System A and System L amino acid transport activity in PHT cells [28]. In line with these observations, we have reported that in placentas of pregnancies in which mTORC1 signaling is lower such as FGR, Nedd4-2 protein expression and SNAT2 and LAT1 ubiquitination are increased and MVM LAT1 and SNAT2 protein expression is decreased [13]. Consistent with these reports, in the current study mTORC1 signaling activity was reduced, Nedd4-2 protein expression increased and ubiquitination of SNAT2 and LAT1 were higher in FGR PHT cells and restoration of mTORC1 signaling by DEPTOR normalization in FGR reversed all these changes. Based on this body of evidence obtained in *ex vivo* human placental samples and in cultured PHT cells, we propose that placental mTORC1 inhibition in pregnancies complicated by FGR results in Nedd4-2 mediated ubiquitination of SNAT2 and LAT1, resulting in decreased amino acid transport to the fetus which contributes to the reduced fetal growth.

We have also previously reported that placental mTORC2 activity and Rac1 and Cdc42 protein expression are decreased in FGR [27]. Additionally, we have demonstrated that mTORC2 signaling stimulates the plasma membrane trafficking of SNAT2 and LAT1 mediated by increasing protein expression of Cdc42 and Rac1, which promote actin polymerization and regulate the actin cytoskeleton [27]. Moreover, Cdc42 silencing decreased System A and System L activity in PHT cells [27], providing evidence that Cdc42 is mechanistically linked to PHT cell amino acid transport capacity. In the current study, we demonstrated that Cdc42 expression was lower, and that the association of F-actin with SNAT2 and LAT1 was decreased in FGR PHT cells. Importantly, restoration of mTOR signaling by silencing *DEPTOR* expression in FGR PHT cells normalized Cdc42 protein expression, SNAT2 and LAT1 isoform association to the F-actin skeleton, the protein expression of SNAT2 and LAT1 in MVM and System A and L transport activity. Collectively, we propose that placental mTORC2 inhibition in FGR results in decreased Cdc42 protein expression, inhibition of SNAT2 and LAT1 association to the actin skeleton and decreased SNAT2 and LAT1 abundance in the trophoblast MVM plasma membrane, resulting in decreased amino acid transport to the fetus which likely contributes to the reduced fetal growth observed in these pregnancies.

We recently reported that trophoblast specific *Mtor* knockdown in mice causes a marked decrease in placental System A and L amino acid transporter activity and fetal growth restriction [12]. Moreover, inhibiting mTOR signaling by gene silencing approaches led to decreased System A and System L amino acid transport activity in cultured PHT cells [15, 49]. FGR PHT cells exhibited decreased expression and activity of System A and System L transporters. On the other hand, activation of mTOR signaling by silencing *DEPTOR* siRNA increases System A and System L amino acid transport in cultured PHT cells isolated from normal pregnancy [27, 28]. Furthermore, using inducible gene targeted approach in mice, Grahammer et al. [50] demonstrated that mTORC1signaling positively influences the expression of the sodium-dependent neutral amino acid transporter (B0 AT1) and cationic amino acid transporter (y+LAT-4F2hc) and regulates the amino acid transport in kidney proximal tubular cells. Jansson and coworkers reported that placental transporters for cationic and neutral amino acids were reduced in syncytiotrophoblast microvillous and basal membrane vesicles isolated from FGR pregnancies [38]. Importantly, reducing DEPTOR signaling in FGR PHT cells restored the System A and L amino acid transport activity in the current study, consistent with the possibility that trophoblast DEPTOR silencing in FGR pregnancy may restore fetal growth by normalizing placental amino acid transport. Consistent with this, we have recently demonstrated in mouse model that trophoblast specific silencing of DEPTOR in the placenta leads to a significant increase in fetal growth, further reinforcing the therapeutic potential of targeting DEPTOR in pregnancies affected by FGR [51, 52].

We have shown that DEPTOR-mTOR signaling regulates fetal facing basal plasma membrane trafficking of LAT1 in FGR PHT cells. System L transporters mediate the efflux of essential amino acids from the syncytiotrophoblast across the BM into the fetal circulation [53]. *In vivo* stable isotope tracer studies have demonstrated reduced transfer of leucine from mother to fetus in human FGR [39]. We have reported a decreased BM System L amino acid transport activity in human FGR [38]. Additionally, Cdc42 silencing decreases the trafficking of LAT1 to the basal plasma membrane of PHT cells [27]. Here we demonstrate that placental Cdc42 expression is decreased in FGR PHT cells and that restoration of Cdc42 by normalization of DEPTOR protein expression in FGR PHT cells, rescues the BM LAT1 expression. Collectively, these data are consistent with the theory that placental mTOR inhibition in FGR contributes to decreased fetal amino acid amino delivery by decreasing the trafficking of LAT1, a key System L isoform, to the fetal-facing BM of the syncytiotrophoblast.

Our findings reveal that hypoxia significantly induces DEPTOR protein expression in PHT cells. This suggests that low oxygen tension characteristic of the FGR placental environment serves as a regulatory stimulus for DEPTOR upregulation, linking hypoxic stress to impaired mTOR signaling and nutrient transport. This novel observation adds mechanistic insight into how environmental stressors modulate placental function. Given that DEPTOR inhibits both mTORC1 and mTORC2, its hypoxia-induced upregulation provides a plausible molecular mechanism for the well-established suppression of placental mTOR activity in FGR. Our findings align with the seminal work of other colleagues, who have demonstrated in both rodent and sheep models that chronic prenatal hypoxia leads to oxidative stress, impaired placental function, and long-term cardiovascular dysregulation in the offspring [50, 54–56]. Recent studies further demonstrate that antioxidant interventions during pregnancy can prevent fetal growth restriction and normalize placental function, underscoring the causal role of hypoxia-induced stress pathways in placental dysfunction [33, 57]. Our data suggests that DEPTOR as a potential mediator of placental dysfunction under hypoxia, offering new avenues for understanding the molecular links between placental stress, mTOR signaling, and fetal growth. Further experiments are needed to delineate the precise molecular pathways through which hypoxia regulates DEPTOR expression and to determine whether this response is mediated directly via canonical hypoxia-inducible factors or alternative stress-responsive signaling mechanisms.

In the current study, we examined the relationship between placental DEPTOR expression and blood pressure in children at 4–6 years of age using longitudinal data from the Healthy Start cohort [36, 58]. Consistent with our prior data from this cohort demonstrated that higher placental mTORC1 signaling was positively associated with birthweight and adiposity both at birth and in early childhood [58], while mTORC2 activation was linked to elevated systolic blood pressure at age 4–6 years [58]. These findings support the concept that placental mTOR activity influences fetal growth and has lasting implications for cardiometabolic health and sustained influence of placental mTOR activity on postnatal metabolic outcomes. Importantly, we now show that placental DEPTOR expression is positively associated with systolic and diastolic blood pressure at 4–6 years of age, providing the first human evidence that upregulation of a negative regulator of mTOR signaling in the placenta may contribute to long-term cardiovascular programming. The precise mechanisms linking placental DEPTOR to offspring blood pressure regulation remain to be established. Together, these findings indicate that placental DEPTOR-mTOR signaling acts as a critical developmental pathway linking *in utero* environment to offspring cardiovascular health. The fact that both low [59] and high birth weights [60] have been independently associated with increased risk of obesity, diabetes, and hypertension further underscores the importance of balanced placental signaling during pregnancy. If our results are replicated in other cohorts and supported by mechanistic studies in animal models, this work could pave the way for targeted placental interventions aimed at preventing fetal programming of chronic disease.

Furthermore, recent findings from the Healthy Start cohort have demonstrated that maternal diet quality, assessed by the Healthy Eating Index (HEI), is associated with sex-specific alterations in placental signaling proteins involved in mTOR pathway [61]. Notably, higher HEI scores were linked to increased phosphorylation of S6K1, a key mTORC1 target in placentas of male offspring, suggesting that maternal nutritional environment can modulate placental mTOR activity [61]. These results complement our current findings by reinforcing the role of placental nutrient sensing pathways in fetal growth regulation. In our study, elevated DEPTOR expression in FGR placentas was associated with suppression of mTOR signaling and impaired amino acid transport. Taken together, these findings support the concept that both intrinsic molecular dysregulation (e.g., DEPTOR-mediated mTOR inhibition) and modifiable maternal factors (e.g., diet quality) converge on placental mTOR signaling to influence fetal growth and postnatal cardiometabolic risk. Future studies should explore whether dietary interventions during pregnancy can modulate DEPTOR expression or its downstream effects on mTOR activity and placental transport function, particularly in pregnancies at risk for FGR.

We recently provided additional evidence that placental DEPTOR expression is negatively correlated to System A amino acid transport activity in human FGR, presenting a novel molecular mechanism for inhibition of mTOR signaling and amino acid transport in human FGR [52]. The molecular mechanisms regulating DEPTOR expression are poorly understood in any cell type, but some reports have implicated Casein kinase I-mediated phosphorylation in the regulation of DEPTOR protein stability and subsequent polyubiquitination by the SCFβ-TrCP E3 ubiquitin ligase for proteasome degradation [62]. Furthermore, transcriptional control of DEPTOR is complex, highly tissue-specific and context-dependent [63]. Our data are consistent with the possibility that increased DEPTOR expression plays an important role in the development of placental dysfunction associated with FGR. In support of this, DEPTOR silencing normalized mTOR signaling, plasma membrane System A and L amino acid transporter expression in FGR PHT cells. However, the molecular mechanisms causing increased trophoblast DEPTOR protein expression in placental insufficiency remain to be fully established.

In summary, this study in primary human trophoblast cells revealed that DEPTOR plays a role in the regulation of mTOR which in turn regulates trophoblast amino acid transporter trafficking and transport activity. We hypothesize that restoration of placental mTOR signaling may represent a novel approach to improve fetal amino acid delivery in pregnancies complicated by FGR with the potential therapeutic goal of increasing fetal growth.

## Materials and methods

### Human placental tissue collection for PHT cells isolation

PHT cells were isolated from placentas collected from term C-section complicated by FGR and term pregnancies delivering appropriate for gestational age (AGA) infants. FGR was defined as an estimated fetal weight (EFW) less than the 5^th^ percentile for gestational age, based on Hadlock growth curves [64] at the most recent ultrasound assessment. Ultrasound estimation of EFW was calculated using measurements of the biparietal diameter, head and abdominal circumference, and femur length according to the Hadlock equation [64]. We confirmed FGR by a birthweight <5^th^ percentile after delivery.

### Ethics approval and consent to participate

All placentas were collected with written informed consent and ethical approval from the Institutional Review Board of the University of Colorado Anschutz Medical Campus (COMIRB 14-1073). All experimental procedures and methods were performed in accordance with the relevant guidelines and regulations, including the World Medical Association Declaration of Helsinki for human studies. Exclusion criteria included smoking, use of illicit drugs, concurrent diseases, such as diabetes and hypertension, and the development of other pregnancy complications including gestational diabetes, pregnancy-induced hypertension, and preeclampsia, fetal anomalies, preterm birth, and birth-related complications.

### Primary human trophoblast cell isolation and culture

Primary human trophoblast cells were isolated from fresh placental villus tissue by trypsin digestion followed by discontinuous Percoll gradient separation as previously described [65]. Trophoblast cells were cultured in either 60 mm culture dishes (∼7.5 ×10^6^ cells/dish for western blot analysis) or 6-well plates (for amino acid uptake experiments; ∼2.75 ×10^6^ cells/well) and cultured in a 1:1 mixture of Dulbecco’s modified Eagle’s medium (DMEM; 25 mM glucose) and Ham’s F-12 medium (10 mM glucose), supplemented with 10% fetal bovine serum, l-glutamine, antibiotics (penicillin, streptomycin, gentamycin). Culture was maintained in 5% CO_2_, 95% atmosphere air at 37 °C for 90 h. Cell culture media was changed daily. The study design, including the time point of siRNA mediated silencing of *DEPTOR* signaling and measurements of various parameters, is illustrated in Supplementary Fig. 1.

### RNA interference-mediated silencing of DEPTOR

Dharmafect 2 transfection reagent (Thermo Scientific, Rockford, IL, USA) and small interference RNAs (siRNAs) (Sigma-Aldrich, St Louis, MO, USA), targeting *DEPTOR* (100 nM; SASI_1297010-H/5582, 1297011-H) and/or a non-coding scrambled sequence (100 nM; sense: 5′GAUCA-UACGUGCGAUCAGATT), were added to cultured primary trophoblast cells (∼2.75 ×10^6^ cells per well in 6-well plate; ∼7.5 ×10^6^ cells in a 60 mm dish) after 18 h in culture, incubated for 24 h, removed, and fresh medium added to the wells [15]. At 90 h in culture, the efficiency of target silencing was determined at the protein and functional levels using western blot.

### System A and System L amino acid uptake assay

System A and System L amino acid transport activity in cultured primary human trophoblast cells was determined at 90 h of culture. The activity of System A transporters was assessed by measuring the Na^+^-dependent uptake of radiolabeled [^14^C] methyl-aminoisobutyric acid (MeAIB; 20 μM), which is a non-metabolizable amino acid analog. System L mediated activity was measured by determining the 2-amino-2-norbornane-carboxylic acid (BCH; 64 μM) inhibitable uptake of [^3^H] leucine (0.0125 μM) [15].

### Isolation of microvillous plasma membranes from trophoblast cells

At 90 hr in culture, trophoblast cells were washed with ice cold PBS and lysed and homogenized in buffer D (10 mM Tris-Hepes, 250 mM sucrose, 1 mM EDTA) with phosphatase and protease inhibitors (1:100, P8340, P2850, P0044, Sigma-Aldrich). Subsequently, microvillous plasma membranes (MVMs) were isolated from cell homogenates of cultured primary human trophoblast cells using differential centrifugation and Mg^2+^ precipitation as described previously [14, 15]. Protein concentration was determined using the Bradford assay (Bio-Rad, CA). The enrichment of alkaline phosphatase, a MVM marker [66], was determined by the ratio of alkaline phosphatase expression in MVM to total cell lysates. Alkaline phosphatase enrichment in MVM isolated from AGA PHT cells transfected with scramble siRNA was 7.15 ± 0.6 (*n* = 6), which was not significantly different from the alkaline phosphatase enrichment in MVM isolated from FGR PHT cells silenced with scramble or *Deptor* siRNA (Supplementary Fig. 2a, b). As an additional enrichment marker, the expression of the insulin receptor β, previously shown to be highly expressed in the MVM of human placenta [67], was determined using Western blot. MVM enrichment of the insulin receptor was not significantly different in the AGA (AGA + Scramble siRNA, 19.3 ± 3.6-fold, *n* = 6) and FGR (FGR + Scramble siRNA, 21.5 ± 3.5-fold; FGR+ *Deptor* siRNA, 20.0 ± 2.7-fold, *n* = 6/each group) silenced PHT cells (Supplementary Fig. 3a, b).

### Isolation of fetal facing basal plasma membranes from trophoblast cells

Basal plasma membranes (BMs) were isolated from cultured PHT cells using a previously described approach [27, 68]. The enrichment of voltage-dependent anion channel (VDAC) [68], a BM marker, was determined by the ratio of VDAC expression in BM to total cell lysates. The VDAC enrichment in BM isolated AGA PHT cells transfected with scramble siRNA was 22.3 ± 2.3-fold (*n* = 6), which was not significantly different from the VDAC enrichment in BM isolated from FGR PHT cells silenced with scramble (20.5 ± 3.7-fold, *n* = 6) or *Deptor* siRNA (20.0 ± 3.8-fold, *n* = 6, Supplementary Fig. 4a, b). These data confirm significant enrichment of the basal plasma membrane from cultured PHT cells.

### Western blotting

For Western Blotting, PHT cells were washed with ice cold PBS. Subsequently, cells were scraped and lysed in radioimmunoprecipitation assay buffer with protease and phosphatase inhibitors, collected and sonicated, snap frozen in liquid N_2_ and stored at −80 °C until further analysis. Proteins in cell lysates and MVM/BMs were separated using Invitrogen electrophoresis system as described previously [14, 15]. The protein abundance and phosphorylation of mTORC1, mTORC2 signaling pathway and DEPTOR expression were determined by Western blot in PHT cell lysates from AGA and FGR PHT cells silenced with scramble or *DEPTOR* siRNA. Total expression and phosphorylation of S6 ribosomal protein (Ser235/236), as a functional readout of mTORC1 signaling, and Akt (Ser473), as a functional readout of mTORC2 signaling were determined using primary antibodies from Cell Signaling Technology, Danvers, MA. DEPTOR protein expression in cell lysates was also determined. Isolated MVM/BM preparations from AGA and FGR PHT cells were used to determine the protein expression of the System A amino acid transporter isoforms SNAT2 and the System L amino acid transporter isoforms LAT1. A polyclonal SNAT2 antibody generated in rabbits was received as a generous gift from Dr P. Prasad at the University of Georgia, Augusta. Antibodies targeting LAT1 were produced in rabbits as described previously [15] and as a generous gift from Yoshikatsu Kanai, Osaka University, Osaka, Japan. The specificity of both SNAT2 and LAT1 antibodies has previously been validated using gene-silencing techniques [9, 10, 69, 70]. A detailed list of primary antibodies used in this study, including target, source, dilution, and catalog information, is provided in Supplementary Table 1. Immunoblots were visualized using enhanced chemiluminescence. Target protein expression was normalized to total protein loaded in the lane. The mean density of the AGA target bands was arbitrarily set to 1 for each protein target. All densitometry values were expressed relative to the AGA mean value.

### Immunoprecipitation and ubiquitination

PHT cell lysates were incubated with SNAT2 or LAT1 or F-actin antibody overnight and antibodies were precipitated with protein G-Sepharose. Immunoprecipitates and aliquots of cell lysates were denatured in sample buffer at 95 °C, resolved by electrophoresis, and probed with ubiquitin or SNAT2 or LAT1 antibody.

### Regulation of DEPTOR by hypoxia in PHT cells

Considering the established role of placental hypoxia in FGR [32], we investigated whether low oxygen tension directly modulates DEPTOR expression in human trophoblast cells. To test this, PHT cells were exposed to defined hypoxic conditions to investigate the regulation of DEPTOR expression in response to oxygen availability. PHT cells were isolated from term placentas of appropriate-for-gestational-age (AGA) pregnancies and cultured at a density of ~5.0 ×10⁶ cells per 60-mm dish in a 1:1 mixture of DMEM (25 mM glucose) and Ham’s F-12 (10 mM glucose), supplemented with 10% fetal bovine serum, l-glutamine, and antibiotics (penicillin, streptomycin, gentamicin). Cells were maintained under standard conditions (5% CO₂, 95% air, 37 °C) for 66 h with daily media changes. After 66 h, cells were washed with PBS and then cultured in fresh medium. Subsequently, cultures were either maintained under normoxic conditions (5% CO₂, 95% air) or exposed to hypoxia (<1% O₂, 5% CO₂, 37 °C) for 24 h [37, 71]. Following treatment, cells were lysed using RIPA buffer supplemented with protease and phosphatase inhibitors for protein analysis. Total protein lysates were used to assess DEPTOR protein expression by Western blotting, as previously described. The study design, including the time point of hypoxia exposure and measurements of other parameters, is illustrated in Supplementary Fig. 5.

### Assessment of DEPTOR expression in the placenta of AGA and SGA pregnancies and its impact on long-term blood pressure outcomes in offspring

#### Longitudinal cohort study and sample collection

To further address the clinical relevance of our findings, we quantified DEPTOR protein expression in placental homogenates from a subset of AGA (*n* = 21) and FGR (*n* = 3) and small-for-gestational age (SGA, *n* = 5) infants of pregnancies enrolled in the Healthy Start study, a prospective longitudinal birth cohort designed to investigate early-life exposures and their impact on child health [36, 37, 58]. We examined placental DEPTOR expression in these samples and their association to cardiovascular outcomes in children at 4–6 years. SGA was defined as a birthweight <10^th^ percentile. FGR was defined as a birthweight <5^th^ percentile. Briefly, placental villus tissue samples from term pregnancies, and excluded participants if they currently had a multiple pregnancy or had a prior history of premature birth, diabetes, or serious psychiatric illness. All participating mothers provided written informed consent, and all procedures were approved by the Colorado Multiple Institutional Review Board (COMIRB). Inclusion criteria for the current analysis required available placental and corresponding child follow-up data at 4–6 years of age. Supplementary Table 2 summarizes the clinical characteristics of the cohort included in this study, grouped by fetal growth status (AGA vs. FGR/SGA).

#### Child blood pressure assessment

Infant birth weight was obtained from medical records. At 4–6 years of age, children returned for a follow-up visit. At this visit, blood pressure was measured with a digital sphygmomanometer.

#### Placenta sample collection and immunoblotting

Trophoblast villus samples were collected after delivery. Samples were snap frozen in liquid nitrogen following collection and stored at −80 °C until further processing. We homogenized ∼20 mg frozen placental villus tissue in 75 μL ice-cold buffer D (250 mmol/L sucrose, 10 mmol/L HEPES, pH 7.4) with a 1:100 dilution of protease and phosphatase inhibitors. Subsequently, we used the ProteinSimple Jess capillary immunoblotting system (SM-PN01-1, Protein Simple, San Jose, CA, USA) as previously reported [72]. Protein abundance was normalized to total capillary protein measured using the total protein detection module (AM-PN01, Protein Simple, San Jose, CA, USA). We used the Jess Simple Western system to quantify DEPTOR protein expression in placental homogenates from AGA and SGA and FGR samples. The mean DEPTOR expression in AGA placentas (*n* = 21) was arbitrarily set to 1.0, and values for FGR (*n* = 3) and SGA (*n* = 5) placentas were expressed relative to this reference.

### Data presentation and statistics

#### PHT cell culture study

GraphPad Prism 10.4.0 software was used to analyze data. Data are presented as mean ± SEM. For PHT cells, the number of experiments (n) represents the number of placentas used to isolate PHT. In amino acid uptake experiments, each condition was studied in triplicate, and data were averaged to represent trophoblast cells isolated from one placenta. The statistical significance of differences between control and experimental groups was assessed using Student’s *t* test. A *P* value < 0.05 was considered significant.

#### Longitudinal cohort study

Pearson’s correlation coefficient (r) was used to evaluate associations between placental DEPTOR expression, birth weight, and child systolic and diastolic blood pressure at 4–6 years of age. Statistical analyses were performed using GraphPad Prism 10.4.0 software, and all data are presented as mean ± SEM. Statistical difference between AGA and SGA group was determined by Student’s *t* test, with significance defined as *P* < 0.05.

## Supplementary information

Supplemental Figures and legends

Supplemental Table 1

Supplemental Table 2

Original data

## Data Availability

All supporting data and associated protocols for this manuscript are available upon request.
