# Supplementary material for: Normalization of trophoblast mTOR signaling rescues impaired function in primary human trophoblast cells isolated from pregnancies complicated by fetal growth restriction
Source: Cell Death Discov. 2025 Nov 7;11:513. doi: 10.1038/s41420-025-02801-5 (PMC12594834; doi:10.1038/s41420-025-02801-5)

**Supplemental Figure 1.**  **Experimental design**. Placentas were collected from the term C-section complicated by FGR and term pregnancies delivering appropriately grown (AGA) infants. Cytotrophoblast cells were isolated from AGA and FGR placentas and cultured for 90 h. PHT cells were transfected with either scramble or *DEPTOR* siRNA at 18 h in culture. Mononuclear cytotrophoblast cells undergo fusion and differentiate into multinucleated syncytiotrophoblast by 72 h in culture and outcomes were studied at 90 h in culture.

**Supplemental Figure 2. MVM enrichment of alkaline phosphatase**. Representative Western blots (a) for alkaline phosphatase in cell lysates and MVM isolated from AGA and FGR PHT cells transfected with either scramble or *DEPTOR siRNA*. A ratio between alkaline phosphatase expression in MVM/ alkaline phosphatase expression in total cell lysates was calculated for each placenta and data is summarized in (b). Values are given as means ± SEM, n=6/each group.

**Supplemental Figure 3. MVM enrichment of insulin receptor β**. Representative Western blots (a) for insulin receptor β in cell lysates and MVM isolated from AGA and FGR PHT cells transfected with either scramble or *DEPTOR siRNA*. The ratio between insulin receptor β expression in MVM/ insulin receptor β expression in homogenates was calculated for each placenta and data is summarized in (b). Values are given as means ± SEM, n=3/each group.

**Supplemental Figure 4. Enrichment of the voltage-dependent anion channel (VDAC) in total cell lysates (TC) in basal plasma membranes (BM).** Representative Western blots (a) for VDAC in cell lysates and BM isolated from AGA and FGR PHT cells transfected with either scramble or *DEPTOR siRNA*. A ratio between VDAC expressions in BM/VDAC expression in total cell lysates was calculated for each placenta and data is summarized in (b). Values are given as means ± SEM.; n=6/each group.

**Supplemental Figure 5. Experimental design**. Placentas were collected from the term C-section pregnancies delivering appropriately grown (AGA) infants. Cytotrophoblast cells were isolated and cultured for 66 h. After 66 hours, cells were washed with PBS and then cultured in fresh medium. Subsequently, cultures were either maintained under normoxic conditions (5% CO₂, 95% air) or exposed to hypoxia (<1% O₂, 5% CO₂, 10% H₂, balance N₂) for 24. Mononuclear cytotrophoblast cells undergo fusion and differentiate into multinucleated syncytiotrophoblast by 72 h in culture and outcomes were studied at 90 h in culture.

**Supplemental Figure 6. Effect of hypoxia on DEPTOR protein expression in PHT cells.** (a) Representative Western blot for DEPTOR in cell lysates of AGA PHT cells exposed to either normoxic conditions (5% CO₂, 95% air) or exposed to hypoxia (<1% O₂, 5% CO₂, 37°C). (b) Histogram summarizes the Western blotting data. Equal loading was performed. After normalization to total protein, the mean density of normoxic samples was assigned to an arbitrary value of 1. Values are given as means + sem. *P < 0.05 vs. AGA PHT cells exposed to normoxic conditions. n=6/each group. paired student t-test.

**Supplemental Figure 7. Associations between birth weight percentile and childhood blood pressure at 4–6 years of age.**

(a) Mean diastolic blood pressure in children born AGA (appropriate-for-gestational-age) compared to those born SGA/FGR (small-for-gestational-age and fetal growth restriction). At 4–6 years of age, children born to pregnancies complicated by FGR or SGA exhibited elevated diastolic blood pressure compared to AGA (control). Diastolic blood pressure was significantly higher in the SGA/FGR group (*p* = 0.01), with an average increase of 8.7% relative to the AGA group. Values are given as means ± SEM.; n=6/each group. P<0.05 vs AGA, student t-Test, n= AGA, 21; FGR, 3 and SGA, 5.

(b) An inverse correlation was observed between birth weight percentile and diastolic blood pressure (p=0.04, r = -0.37), suggesting that lower birth weight is associated with elevated diastolic blood pressure in early childhood. r=Pearson correlation coefficient, n= AGA, 21; FGR, 3 and SGA, 5.

(c) Mean systolic blood pressure in children born with AGA versus those born SGA/FGR. Systolic blood pressure also trended higher in the SGA/FGR group (*p*= 0.09), corresponding to a 5% increase compared to AGA. Values are given as means ± SEM.; n=6/each group. P<0.05 vs AGA, student t-Test, n= AGA, 21; FGR, 3 and SGA, 5.

(d) An inverse correlation was observed between birth weight percentile and systolic blood pressure (p=0.04, r = -0.38), suggesting that lower birth weight is associated with elevated diastolic blood pressure in early childhood. r=Pearson correlation coefficient, n= AGA, 21; FGR, 3 and SGA, 5.


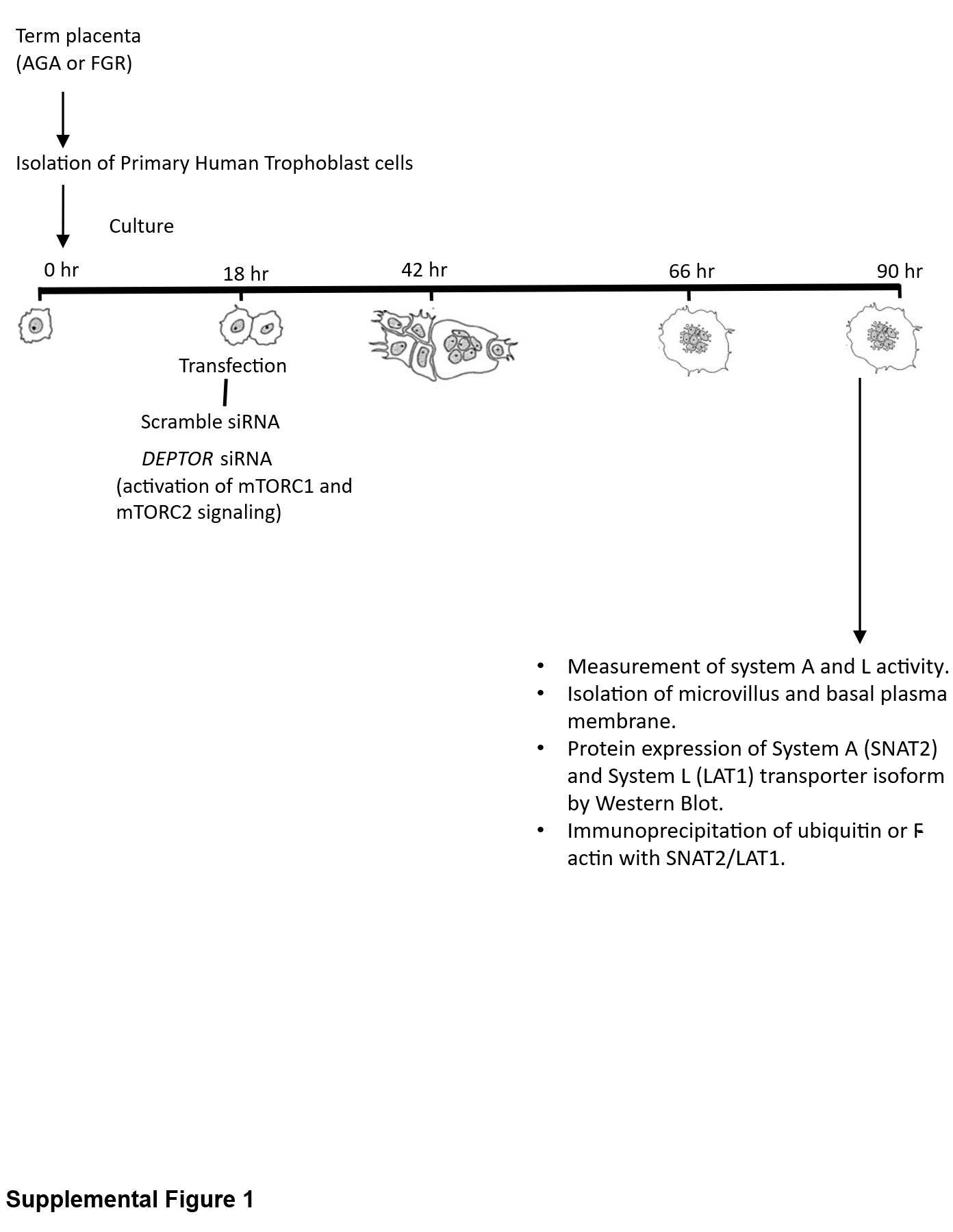


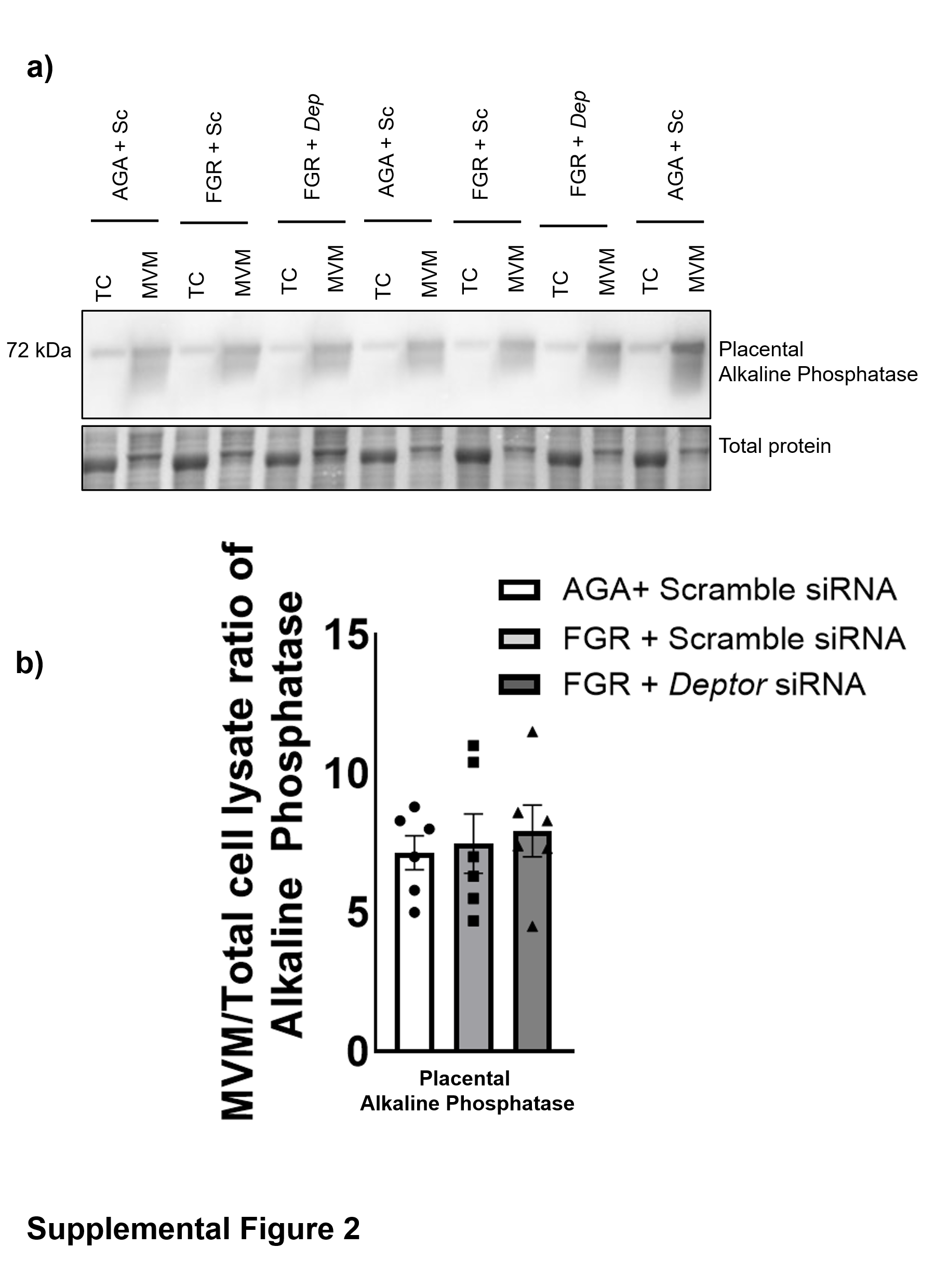


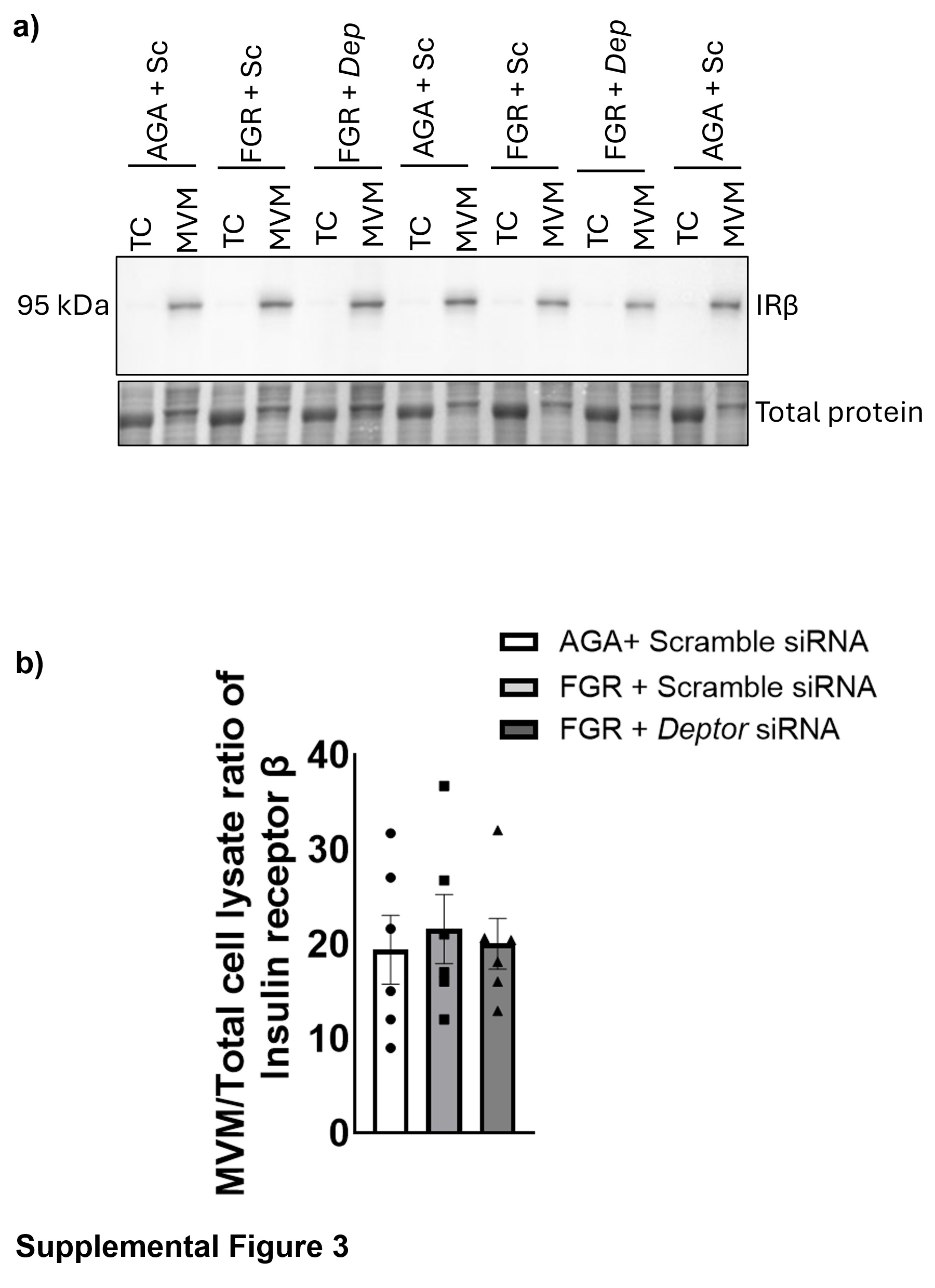


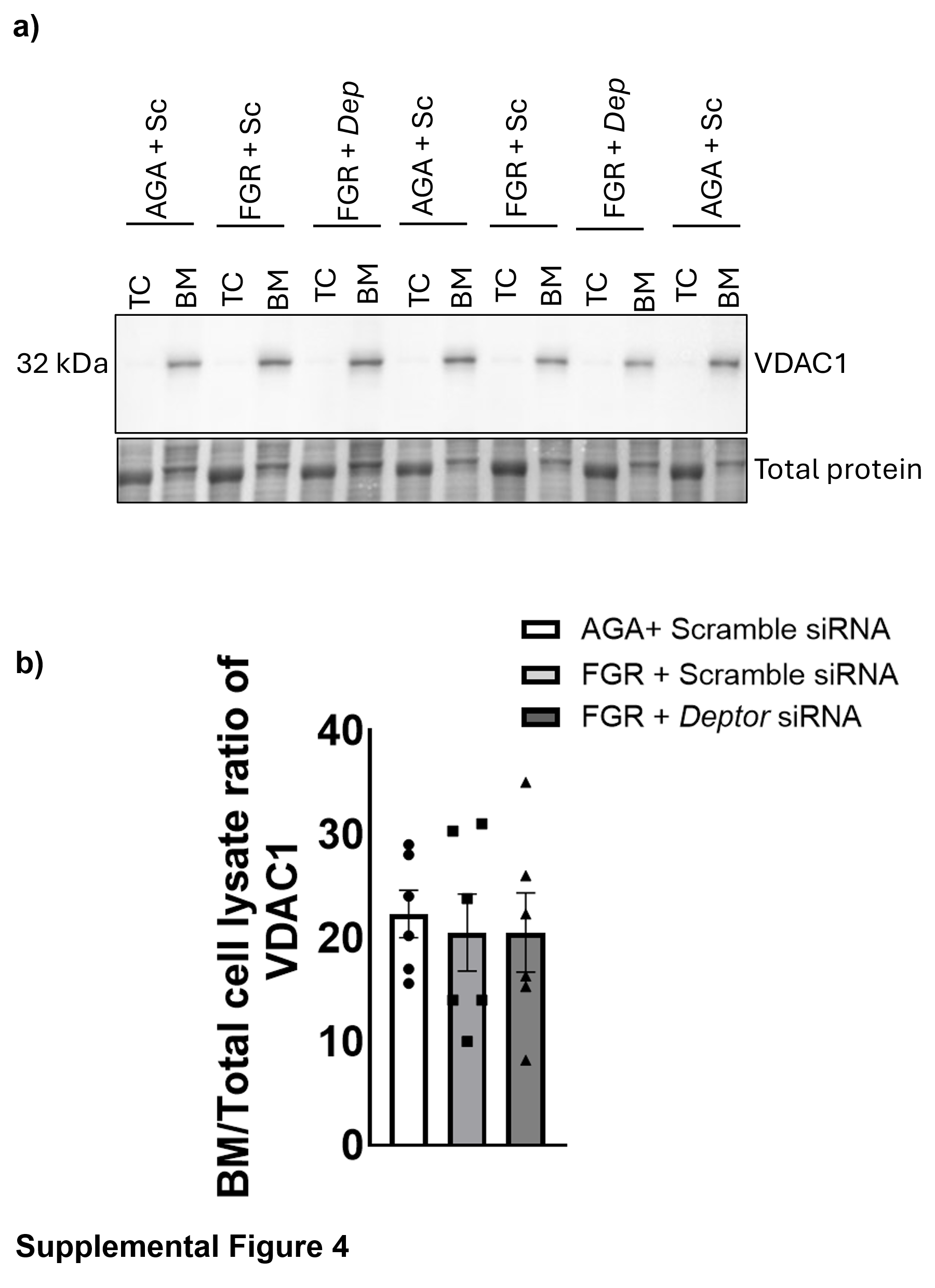


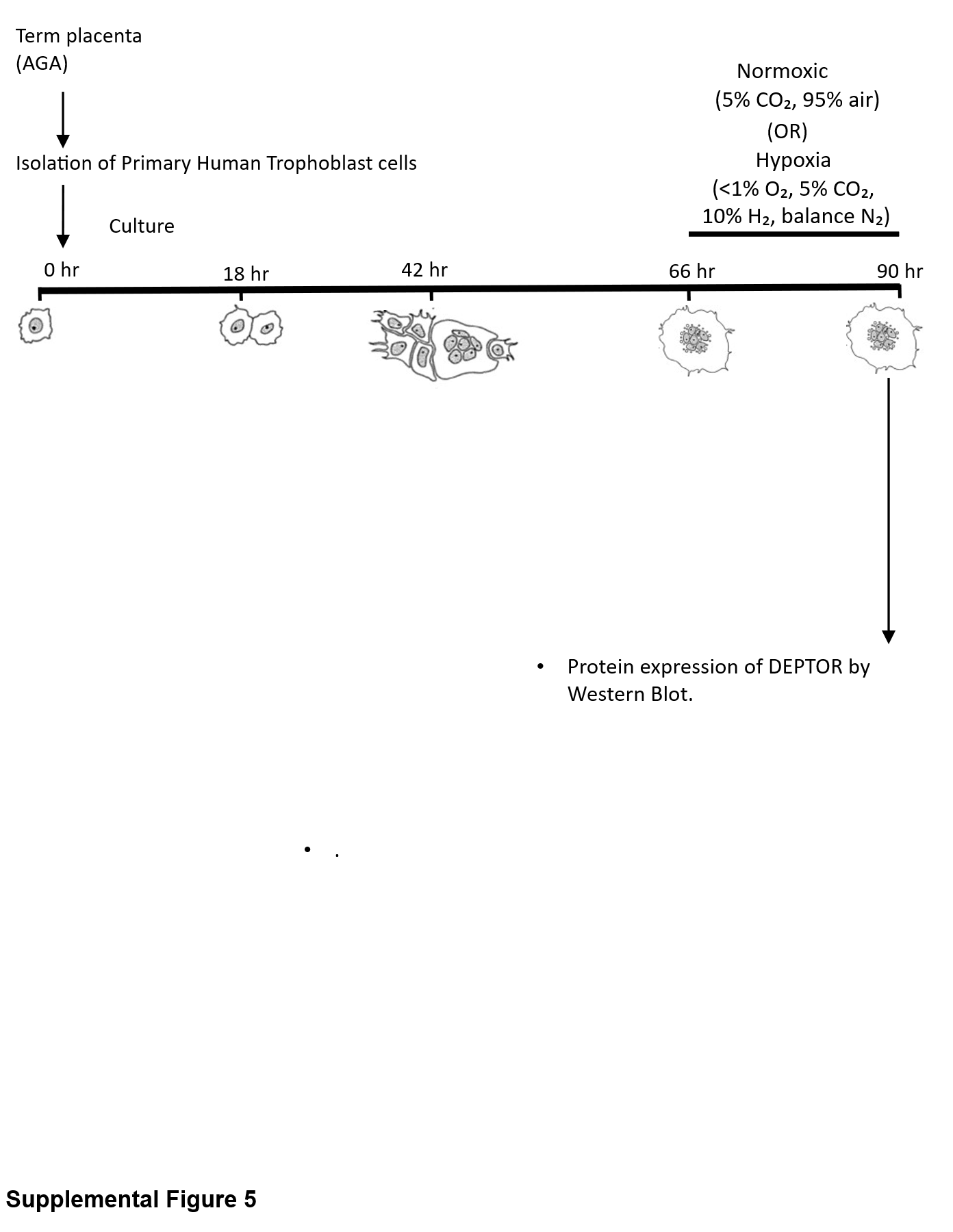


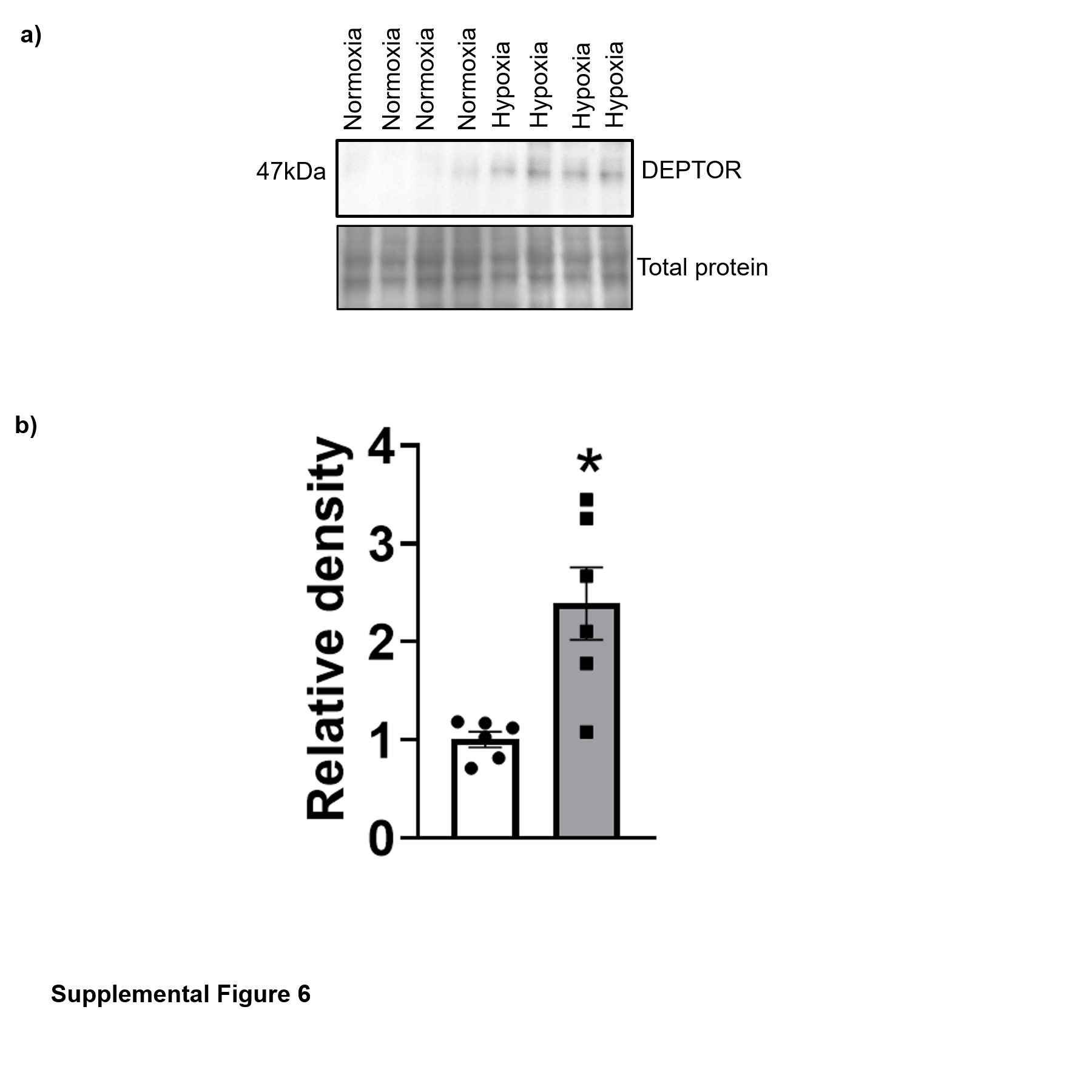


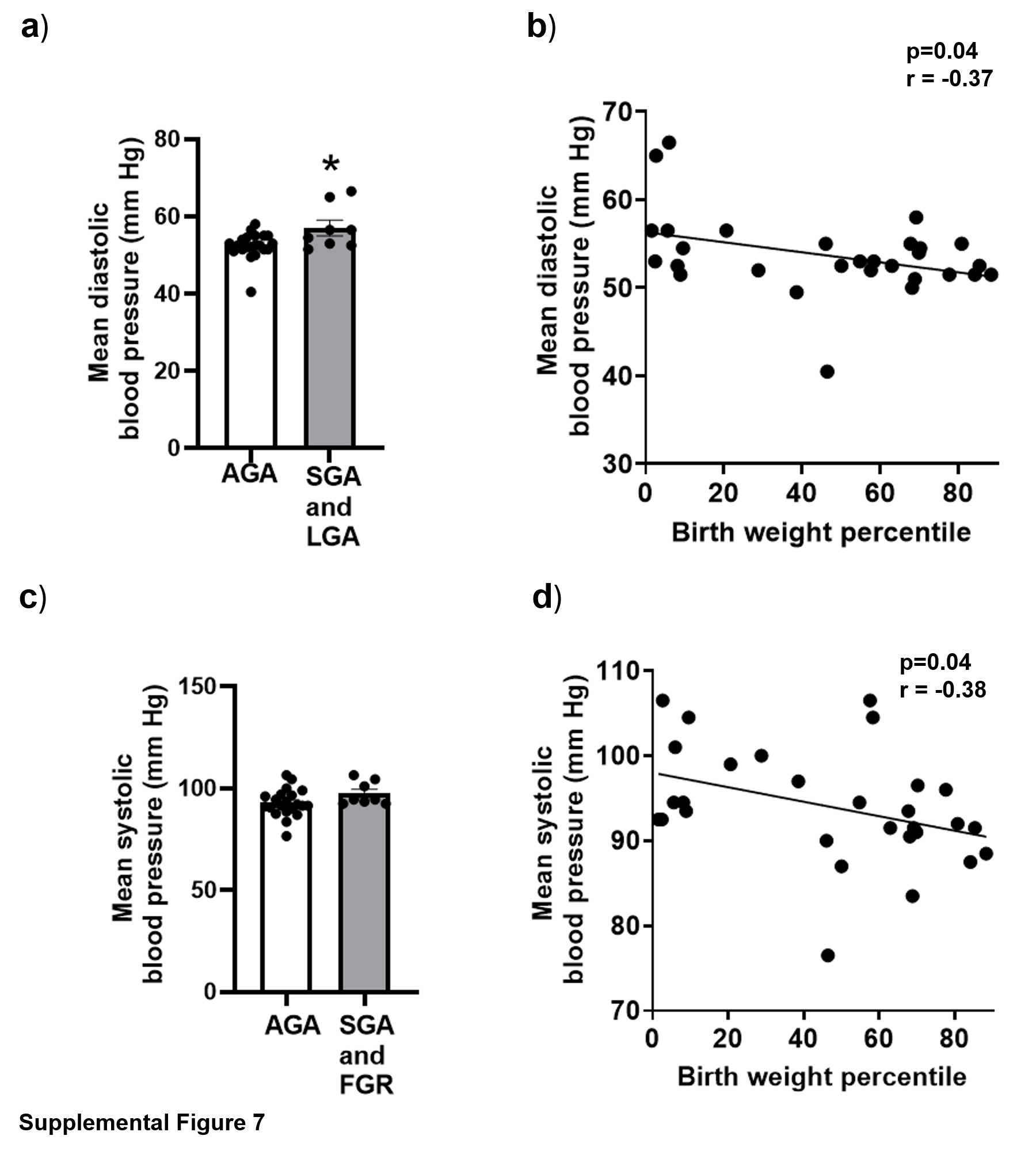

Supplement: Supplementary file 1 — Supplemental Figures and legends [file 41420_2025_2801_MOESM1_ESM.docx]
