# Supplementary material for: Normalization of trophoblast mTOR signaling rescues impaired function in primary human trophoblast cells isolated from pregnancies complicated by fetal growth restriction
Source: Cell Death Discov. 2025 Nov 7;11:513. doi: 10.1038/s41420-025-02801-5 (PMC12594834; doi:10.1038/s41420-025-02801-5)
Supplement: Supplementary file 2 — Supplemental Table 1 [file 41420_2025_2801_MOESM2_ESM.docx]

**Supplemental Table 1. List of primary and secondary antibodies**

| Target | Species | Dilution of primary antibody | Dilution of secondary antibody | Manufacturer | Catalogue number |
| --- | --- | --- | --- | --- | --- |
| S6RP (Ser-235/236) | rabbit | 1:1000 | 1:3000 | Cell Signaling | #2211 |
| S6RP | rabbit | 1:1000 | 1:3000 | Cell Signaling | #2217 |
| AKT (Ser-473) | rabbit | 1:500 | 1:3000 | Cell Signaling | #9271 |
| AKT | rabbit | 1:500 | 1:3000 | Cell Signaling | #9272 |
| Deptor | rabbit | 1:1000 | 1:3000 | Novus Biologicals | NBP1-49674SS |
| SNAT2 | rabbit | 1:5000 | 1:1000 | Gift from Dr P. Prasad^1^ |  |
| LAT1 | rabbit | 1:5000 | 1:1000 | Gift from Dr K. Kanai^2^ |  |
| Cdc 42 | rabbit | 1:1000 | 1:3000 | Cell Signaling | #2466 |
| Nedd4-2 | rabbit | 1:1000 | 1:3000 | abcam | ab46521 |
| VDAC | rabbit | 1:1000 | 1:3000 | abcam | ab15895 |
| PLAP | rabbit | 1:1000 | 1:3000 | abcam | ab133602 |
| IR-β | rabbit | 1:1000 | 1:3000 | Cell Signaling | #3025 |
| β-actin | rabbit | 1:1000 | 1:3000 | Cell Signaling | #4970 |

Abbreviations: S6RP, S6 ribosomal protein; Ser, Serine; AKT, protein kinase B; SNAT2, Sodium-coupled neutral amino acid transporter 2; LAT1, L-type amino acid transporter 1; Cdc 42, cell division control protein 42; Nedd4-2, neural precursor cell–expressed developmentally down-regulated 4-2; VDAC, voltage-dependent anion channel; PLAP. placental alkaline phosphatase; IR-β, insulin receptor-beta.

1 Ling, R. *et al.* Involvement of transporter recruitment as well as gene expression in the substrate-induced adaptive regulation of amino acid transport system A. *Biochim Biophys Acta* **1512**, 15-21 (2001). <https://doi.org/10.1016/s0005-2736(01)00310-8>

2 Park, S. Y. *et al.* Reabsorption of neutral amino acids mediated by amino acid transporter LAT2 and TAT1 in the basolateral membrane of proximal tubule. *Arch Pharm Res* **28**, 421-432 (2005).
