# Supplementary material for: Normalization of trophoblast mTOR signaling rescues impaired function in primary human trophoblast cells isolated from pregnancies complicated by fetal growth restriction
Source: Cell Death Discov. 2025 Nov 7;11:513. doi: 10.1038/s41420-025-02801-5 (PMC12594834; doi:10.1038/s41420-025-02801-5)
Supplement: Supplementary file 3 — Supplemental Table 2 [file 41420_2025_2801_MOESM3_ESM.docx]

**Supplemental Table 2**: Clinical characteristics of longitudinal cohort study samples

|  | AGA | SGA/FGR | P value |
| --- | --- | --- | --- |
| BMI (kg/m^2^) | 24.98 ± 1.104 (17.98-36.27) | 24.40 ± 1.102 (19.36-28.84) | 0.19 |
| Gestational age (weeks) | 39.90 ± 0.29 (37.71-42.14) | 38.31 ± 0.49 (37.14-39.43) | 0.015 |
| Birth weight (g) | 3419.00 ± 63.67  (2830-4025) | 2438.00 ± 56.45  (2200-2720) | <0.0001 |
| Birth weight percentile (%) | 61.62+4.00 (20.61-88.31) | 5.56+1.10 (1.50-9.51) | <0.0001 |
| sex (male/female) | 8/13 | 5/3 | 0.41 |

Values are presented as mean ± SEM.

Statistical significance of differences between AGA and FGR/SGA groups was assessed using unpaired Student’s t-test or Fisher’s test.
