# Supplementary material for: Normalization of trophoblast mTOR signaling rescues impaired function in primary human trophoblast cells isolated from pregnancies complicated by fetal growth restriction
Source: Cell Death Discov. 2025 Nov 7;11:513. doi: 10.1038/s41420-025-02801-5 (PMC12594834; doi:10.1038/s41420-025-02801-5)

Figure 1

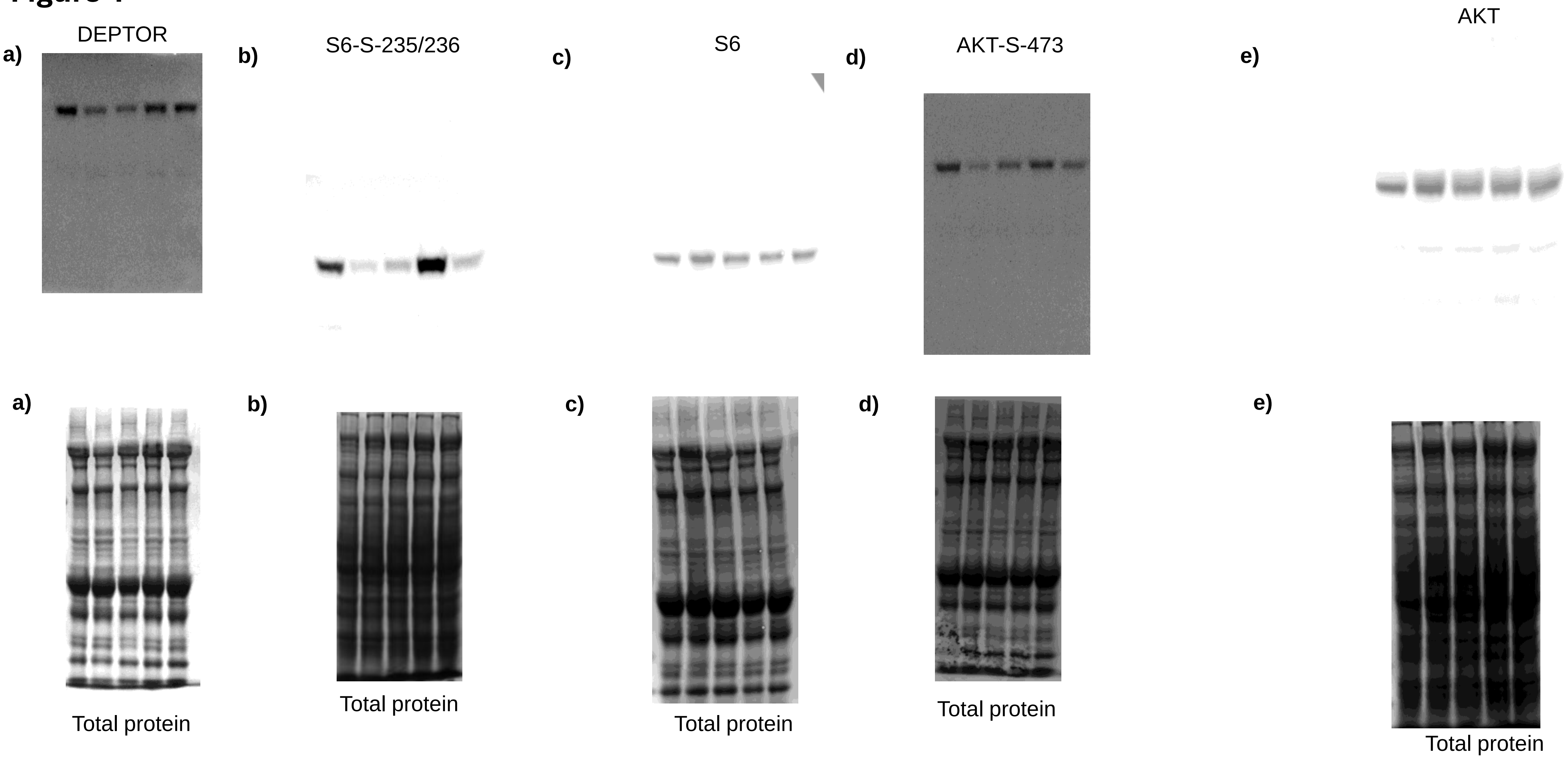
a)
DEPTOR
b)
S6-S-235/236
c)
S6
d)
AKT-S-473
e)
AKT
a)
Total protein
b)
Total protein
c)
Total protein
d)
Total protein
e)
Total protein

Figure 2

DEPTOR Total protein

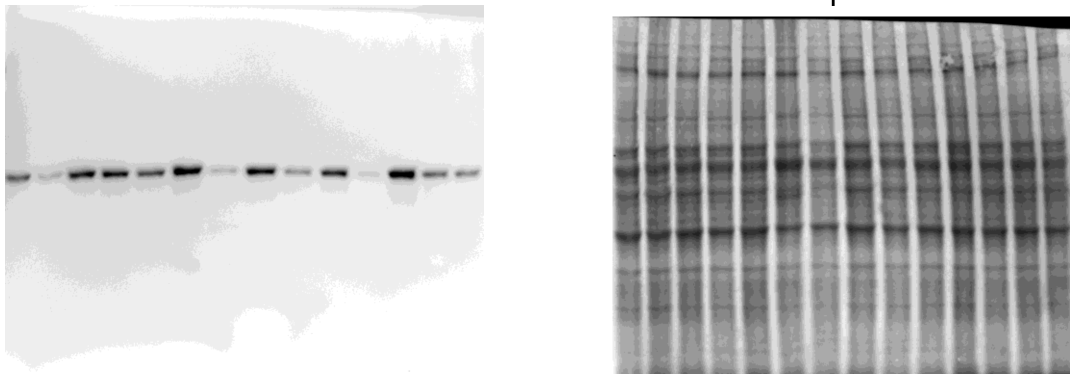

Figure 3

a)

S6-S-235/236

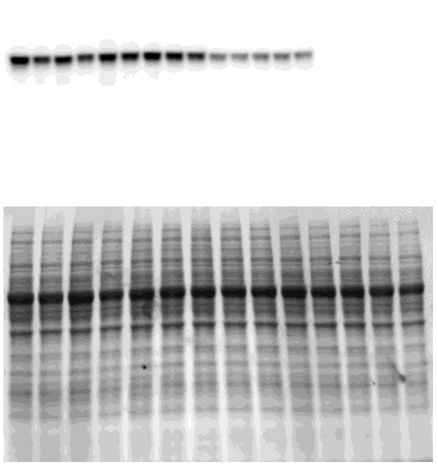

Total protein

b)

S6

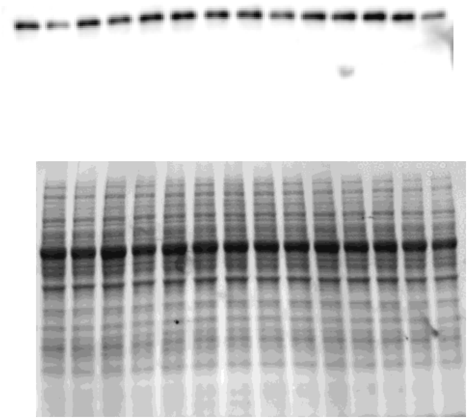

Total protein

Figure 4

a)

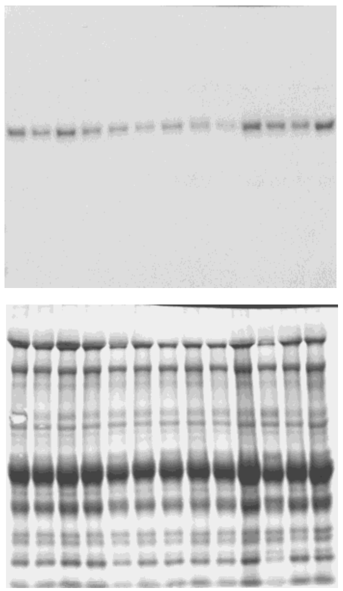

b)

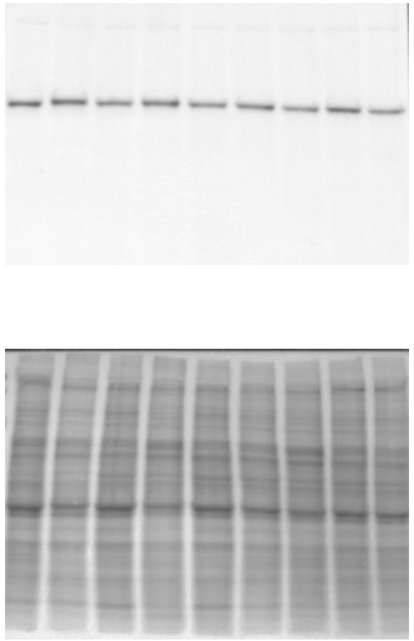

Figure 5

SNAT2

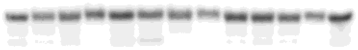

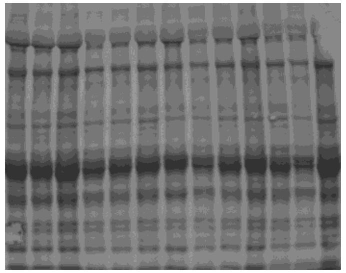

Total protein

f)

LAT1

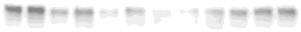

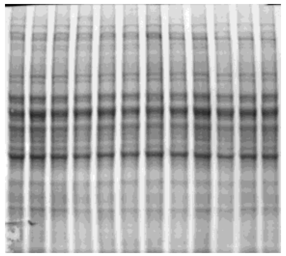

Total protein

Figure 6

a)

Nedd4-2

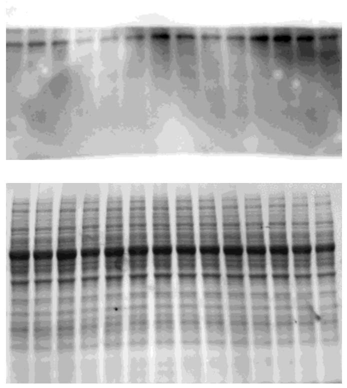

Total protein

c)

SNAT2-ubiqutin

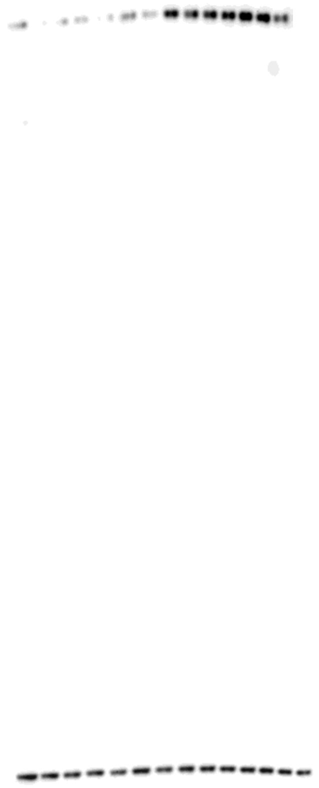

SNAT2

e)

LAT1-ubiqutin

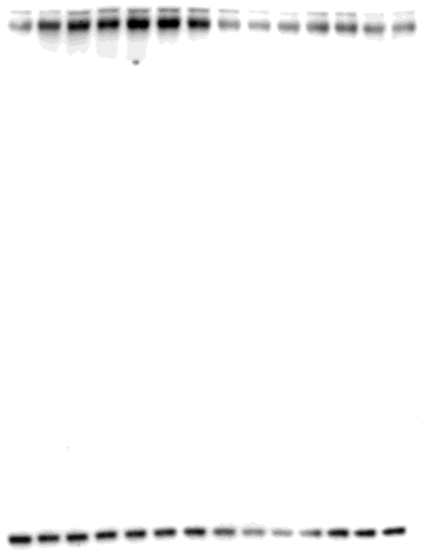

LAT1

Figure 7

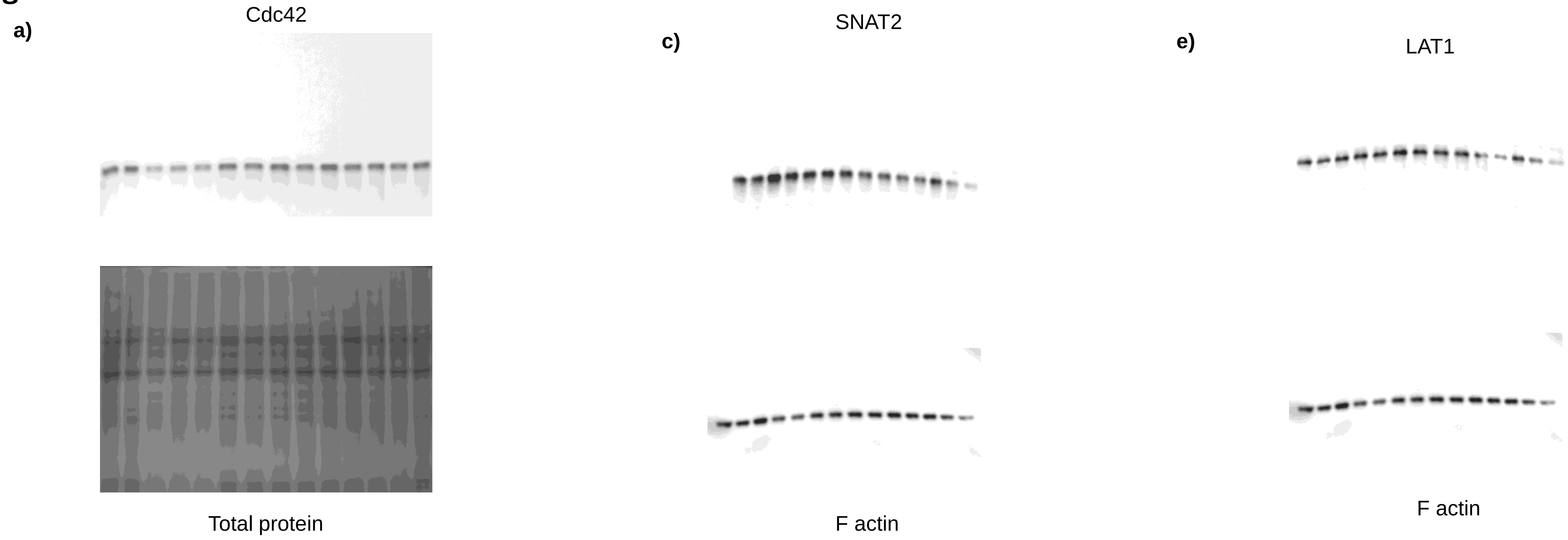

Figure 8

a)

LAT1

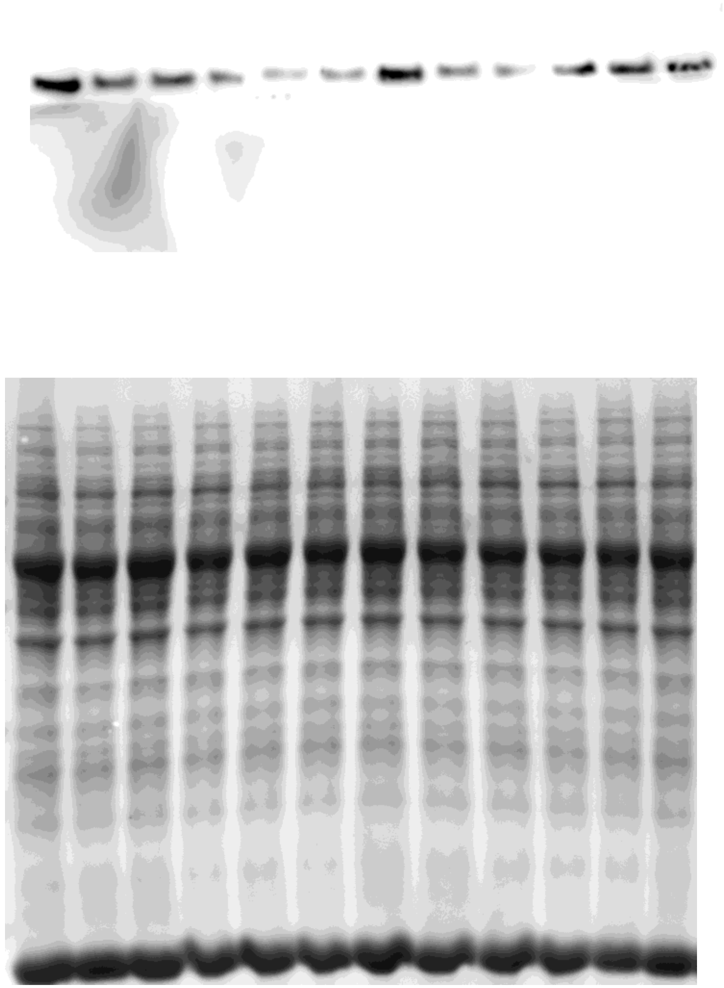

Total protein

Supplemental Figure 2

Alkaline phosphatase

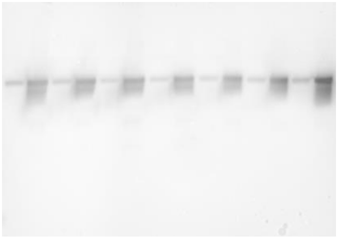

Total protein

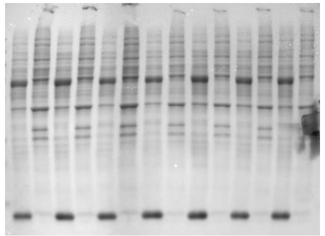

Supplemental Figure 3

IR beta

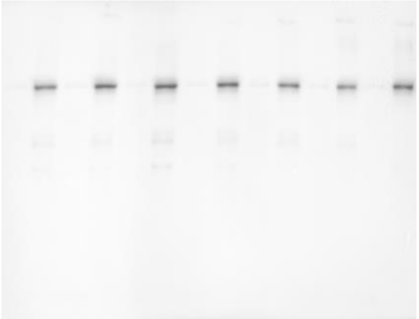

Total protein

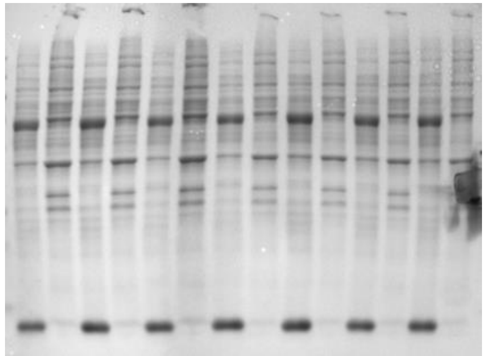

VDAC1

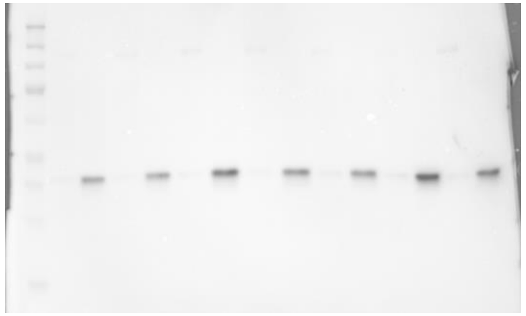

Total protein

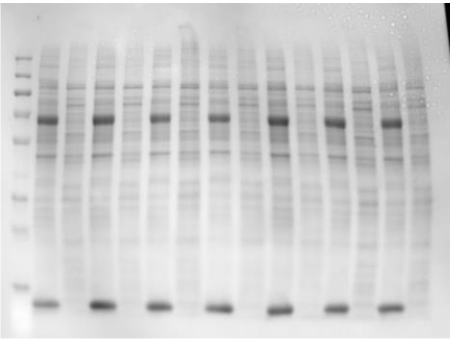

Supplement: Supplementary file 4 — Original data [file 41420_2025_2801_MOESM4_ESM.pdf]
